# Changes in hemoglobin levels and cardiometabolic health in adults with metabolic syndrome – a secondary outcome analysis of a six-month randomized controlled trial

**DOI:** 10.1080/07853890.2026.2635205

**Published:** 2026-02-28

**Authors:** Jasmina Gassner, Jooa Norha, Tanja Sjöros, Taru Garthwaite, Tiia Koivula, Saara Laine, Mikko Koivumäki, Henri Vähä-Ypyä, Petri Kallio, Maria Saarenhovi, Eliisa Löyttyniemi, Harri Sievänen, Noora Houttu, Kirsi Laitinen, Kari K. Kalliokoski, Tommi Vasankari, Peppi Koivunen, Juhani Knuuti, Ilkka Heinonen

**Affiliations:** ^a^ Turku PET Centre, University of Turku, Åbo Akademi University, and Turku University Hospital, Turku, Finland; ^b^ The UKK Institute for Health Promotion Research, Tampere, Finland; ^c^ Department of Clinical Physiology and Nuclear Medicine, University of Turku and Turku University Hospital, Turku, Finland; ^d^ Paavo Nurmi Centre and Unit for Health and Physical Activity, University of Turku, Turku, Finland; ^e^ Department of Biostatistics, University of Turku and Turku University Hospital, Turku, Finland; ^f^ Institute of Biomedicine and Food and Nutrition Research Center, University of Turku, Turku, Finland; ^g^ Department of Biomedical Engineering, Huazhong University of Science and Technology, Wuhan, Hubei, China; ^h^ Faculty of Medicine and Health Technology, Tampere University, Tampere, Finland; ^i^ Biocenter Oulu and Faculty of Biochemistry and Molecular Medicine, Oulu Center for Cell-Matrix Research, University of Oulu, Oulu, Finland

**Keywords:** Hemoglobin, insulin sensitivity, metabolic syndrome, sedentary behavior, physical activity, hyperinsulinemic-euglycemic clamp

## Abstract

**Background:**

Lower hemoglobin (Hb) levels within the normal range have been associated with favorable metabolic traits in cross-sectional studies. This study investigated whether changes in Hb levels correlated with changes in physiological and cardiometabolic parameters during a six-month behavioral intervention in individuals with metabolic syndrome.

**Methods:**

The six-month randomized controlled trial aimed to reduce sedentary behavior in adults with metabolic syndrome (*n* = 64). Key measurements included fasting blood samples, insulin sensitivity during a hyperinsulinemic-euglycemic clamp, insulin-stimulated liver glucose uptake, liver fat content (LFC), indirect calorimetry, cardiorespiratory fitness, and cardiac function. Correlations between changes in these variables and changes in Hb levels at baseline, three, and six months were examined.

**Results:**

Cross-sectionally, higher Hb levels correlated with lower insulin sensitivity (r=-0.35, *p* = 0.005), higher resting O_2_ consumption (*r* = 0.41, *p* < 0.001), higher resting energy expenditure (*r* = 0.49, *p* < 0.001), higher LFC (*r* = 0.40, *p* = 0.011), and greater left ventricular wall thickness (*r* = 0.42, *p* = 0.001). The intervention did not significantly impact Hb levels, and changes in Hb levels did not correlate with most cardiometabolic changes. However, reduced Hb levels correlated with reduced fasting blood glucose (*r* = 0.29, *p* = 0.032), improved insulin sensitivity (r = -0.26, *p* = 0.045), and increased cardiorespiratory fitness (r = -0.29, *p* = 0.033).

**Conclusions:**

Changes in Hb levels did not consistently correlate with changes in cardiometabolic markers during the intervention. However, reductions in Hb levels may relate to improved insulin sensitivity and fitness. Along cross-sectional correlations, this may be clinically relevant for individuals with metabolic syndrome. Further studies are merited to clarify the role of Hb levels in this high-risk group.

## Graphical Abstract

**Figure:**
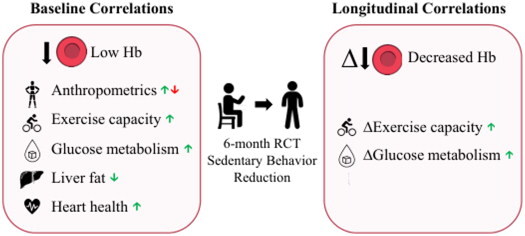

## Introduction

Red blood cells (RBCs) and their hemoglobin (Hb) are essential components for transporting oxygen to peripheral tissues in the human body and therefore play a crucial part in energy metabolism. This process is impaired in anemia, when Hb levels are below the normal range. However, many studies have shown that within the normal reference values (134–167 and 117–155 g/l for Finnish men and women, respectively) [1], lower Hb is beneficially and higher Hb detrimentally associated with health [2–15]. In particular, higher Hb levels have been associated with increased body adiposity and metabolic derangements, such as impaired glucose tolerance, insulin resistance, and dyslipidemia [2–6]. Those metabolic derangements are key features of metabolic syndrome and contribute to increased cardiometabolic risk [16]. Consistent with this, evidence shows that higher Hb levels are associated with a higher prevalence [15] and incidence [17] of metabolic syndrome and its components [2,4,6,15]. In addition, higher Hb levels have been associated with larger retinal vascular caliber [7], worse periodontal status [8], a higher risk of gestational diabetes [9,10], lower baroreflex sensitivity and heart rate variability [12], increased large artery stiffness [18], cardiac dysfunction [11,13], a higher prevalence of polycystic ovary syndrome [14], and increased oxygen consumption at rest [2]. However, most of these associations are based on cross-sectional population studies which cannot establish causality. A Finnish study of 967 adults investigated the prognostic value of Hb levels and revealed that individuals with Hb levels in the highest quartile had increased overall and cardiovascular-related mortality rates over a 20-year follow-up period [15].

While there is a growing body of evidence that elevated Hb levels are linked to worse cardiometabolic health and worse survival, the causality behind these associations remains unclear. Recent evidence suggests a potential mediation by tissue oxygenation and the hypoxia-inducible factor pathway [2] but it has not yet been studied if within-individual changes in Hb levels are associated with changes in cardiometabolic markers over time.

We have performed a six-month randomized controlled trial aimed at a sedentary behavior (SB) reduction in adults with metabolic syndrome and overweight or obesity [19–26]. Both the intervention and control groups showed minor improvements in body weight and composition [25]. Although the intervention was not primarily aimed at reducing Hb levels, it provided a controlled setting with comprehensive repeated measurements, allowing us to examine the relationship between changes in Hb levels and markers of cardiometabolic health. This approach can yield valuable information beyond cross-sectional analyses, even in the absence of significant differences between groups. Measurements included body composition, comprehensive blood analyses, whole body insulin sensitivity during hyperinsulinemic-euglycemic clamp (HEC), liver insulin sensitivity using [^18^F]fluoro-deoxy-glucose positron emission tomography ([^18^F]FDG-PET) and liver fat content based on magnetic resonance spectroscopy (MRS), resting energy consumption and metabolic flexibility by calorimetry, maximal aerobic fitness, echocardiographic measures, self-reported dietary assessments, and accelerometer-measured physical activity (PA) and SB, and the associations of all above-mentioned with Hb levels and other RBC-related measures based on blood count measured at baseline, three-month, and six-month time points.

The rationale behind the present study was that if a causal relationship existed, Hb levels, body adiposity, and the associated cardiometabolic derangements would correlate with each other during the intervention. From a clinical perspective, it is important to better understand the connection between Hb levels and metabolic function. Hb levels are routine measurements and clarifying their role could have implications for risk stratification and monitoring of cardiometabolic health, especially in individuals at high-risk, such as those with metabolic syndrome.

We hypothesized that changes in Hb levels during the intervention would correlate with changes in body adiposity and cardiometabolic derangements.

## Methods

In this study, data from a randomized controlled trial (RCT) conducted at the Turku PET Centre, Turku, Finland was used for secondary analysis. The parallel-group RCT aimed to reduce sedentary time in free-living conditions over a six-month period, with the primary endpoint being whole-body insulin sensitivity. The RCT was conducted between 04/2017 and 03/2020, was approved by the Ethics Committee of the Hospital District of Southwest Finland (16/1801/2017), and adhered to the principles of the Declaration of Helsinki (version 2013). The study was pre-registered at ClinicalTrials.gov (NCT03101228, April 5, 2017). Prior to the enrolment, written informed consent was obtained from all participating individuals.

### Study participants

The recruitment process and inclusion and exclusion criteria have been described in detail in previous publications [25,27]. Briefly, participants volunteered, and the inclusion criteria were age of 40–65 years, body mass index (BMI) of 25–40 kg/m^2^, metabolic syndrome [28], less than 120 min of self-reported moderate-intensity PA per week, more than 10 h or 60% of accelerometer wear time of SB per day measured during a four-week screening period. Exclusion criteria were diagnosis of diabetes, previous cardiac events, depression or bipolar disorder, abundant alcohol use, use of narcotics or tobacco, and any condition that would put the participant at risk or interfere with the study. Sample size calculation for the primary outcome of the RCT (whole-body insulin sensitivity) resulted in the recruitment of sixty-four participants [25].

### Intervention

The six-month intervention phase was preceded by a one-month screening phase, after which randomization into intervention and control groups was performed by a statistician using SAS 9.4 (SAS Institute Inc., Cary, NC, USA) using random permuted block randomization in a 1:1 ratio for men and women separately.

As previously reported [25], individuals in the intervention group had the goal of reducing their daily SB by one hour for six months from their baseline SB measured during the screening month. SB was replaced by standing, light physical activity (LPA) and moderate-to-vigorous physical activity (MVPA) (maximum 20 min), based on participants’ preferences discussed during an individual counselling session. No increase to any physical exercise training was encouraged, but daily non-exercise activities, such as using standing desks or walking when on the phone, were recommended for replacing SB. The control group was advised to maintain their usual behavior. All participants used accelerometers connected to a mobile phone application (ExSed, UKK Terveyspalvelut Oy, Tampere, Finland) allowing them to self-monitor their PA behavior. Additionally, the individualized goals for each PA intensity and SB were set on the application to facilitate adherence to the intervention or control.

### Measurements

Accelerometers were used continuously during the one-month screening period and the six-month intervention period. Other measures were assessed at baseline (after the screening), after the six-month intervention, and partially at three months (anthropometrics, blood parameters).

### Blood variables

To measure RBC variables (Hb, RBC count, hematocrit (Hct), mean corpuscular volume (MCV), mean corpuscular hemoglobin (MCH)), glucose concentration, insulin, HbA1c, blood lipids (triglycerides, low density lipoprotein (LDL), high density lipoprotein (HDL) and total cholesterol), and liver enzymes (alanine aminotransferase (ALT), aspartate aminotransferase (AST), γ-glutamyltransferase (GGT)) venous blood samples were drawn after ≥10h of fasting and analyzed at the Turku University Hospital Laboratory, as specified before [3,25,29,30].

### Anthropometrics

After ≥ four hours of fasting, body weight, fat mass, fat-free mass (FFM), and body fat percentage were assessed by air displacement plethysmography (Bod Pod, COSMED USA Inc., Concord, CA, USA). Height was measured with a wall-mounted stadiometer, and waist circumference was measured with a tape measure at the midpoint between the iliac crest and the lowest rib.

### Sedentary behavior and physical activity

As described previously [25], to assess SB and PA, participants wore triaxial accelerometers at the hip during waking hours during both the screening (UKK AM30, UKK Terveyspalvelut, Tampere, Finland) and intervention periods (Movesense, Suunto, Vantaa, Finland). Wear time of 10-19h/day was considered valid. Data was analyzed in epochs of 6 s using the mean amplitude deviation method to convert the raw acceleration data to metabolic equivalents (METs) [31]. Based on this, LPA (1.5 to <3.0 METs) and MVPA (≥3METs) were recognized. The angle for posture estimation method was used for epochs with MET values below 1.5 to distinguish standing from SB [31,32].

### Cardiorespiratory fitness

As reported in a previous study [22], a graded maximal exercise test was performed on a recumbent cycle ergometer (eBike EL Ergometer and CASE v6.7, GE Medical Systems Information Technologies Inc., Milwaukee, WI, USA). Starting with 25 W, the load was increased in increments of 25 W every three min until exhaustion or a medical reason for termination. Maximal oxygen uptake VO_2max_ (mL/kg/min), VO_2max_ per FFM (mL/kg_FFM_/min), and maximal power output per body mass (W_max_/kg) and FFM (W_max_/kg_FFM_) were assessed.

### Dietary intake

Participants kept four-day food diaries (including one weekend day) to assess dietary intake, both before and at the end of the intervention period. A qualified researcher assessed total daily energy intake and intake of carbohydrates, proteins, total fat, saturated fatty acids, monounsaturated fatty acids, and polyunsaturated fatty acids using computerized software (AivoDiet, Aivo, Turku) based on the Finnish Food Composition Database, Fineli (Fineli, https://fineli.fi/fineli/en/).

### Hyperinsulinemic-euglycemic clamp

To assess insulin sensitivity, hyperinsulinemic-euglycemic clamp (HEC) was performed after an overnight fast and according to a previously described protocol [25]. Briefly, after priming with higher doses of insulin, an insulin infusion was administered at a continuous rate of 40 mU/min/m^2^ body surface area. After four min, a 20% glucose infusion was started and adjusted every five to 10 min to maintain a blood glucose level of approximately five mmol/L. The M-value, defined as whole-body glucose uptake in micromoles per kilogram of body mass per minute, was calculated from steady-state glucose values and glucose infusion rate values beginning 20 min after starting the clamp.

### Indirect calorimetry

As previously reported [33], a ventilated hood system (Quark RMR + OMNIA, COSMED, Rome, Italy) was used for indirect calorimetry at rest and during insulin simulation with HEC. Resting values were determined for 20 (SD 2) min after overnight fasting. In addition, respiratory gas exchange was assessed for 15 (SD 2) min during HEC, starting at 29 (SD 8) min, as well as breath-by-breath throughout the cycle ergometer test with a mask (Vyntus CPX, CareFusion, Yorba Linda, CA, USA). The obtained VO_2_ and VCO_2_ values were used to calculate resting energy expenditure (kcal/d) and metabolic flexibility, defined as the change in respiratory exchange ratio (RER = VCO_2_/VO_2_), from fasting to insulin-stimulation and from low- to maximal-intensity exercise.

### Liver measurements

The measurement of liver glucose uptake (LGU), endogenous glucose production (EGP), and liver fat content (LFC) was described earlier in more detail [34–36]. LGU was measured during HEC, using [^18^F]FDG PET/CT imaging (GE D690, GE Healthcare, Milwaukee, US). [^18^F]FDG was produced [37] and its uptake analyzed according to previously published methods [38]. 168 (SD 11) MBq of the tracer was injected in the antecubital vein 75 (SD 12) minutes after clamp start, and dynamic hepatic imaging was started at time of injection. Tracer availability was derived from radioactivity in the left ventricle and blood samples. To measure [^18^F]FDG activity, a region of interest was drawn in the right liver lobe using CT for anatomical reference. Technical correction and iterative reconstruction were done, and data was analyzed with Carimas software (v2.71, Turku PET Centre, Turku, Finland). LGU (µmol/ml/min) was calculated as Ki (tissue fractional phosphorylation rate) * average plasma glucose, divided by the liver lumped constant of 1.0. For plasma radioactivity an automatic gamma counter (Wizard 1480 3ʺ, Wallac, Turku, Finland) was used.

As previously described [35], EGP was calculated as the difference between the glucose rate of disappearance and the space-corrected glucose infusion rate during HEC.

LFC measurement, as described [34–36] was done with magnetic resonance spectroscopy (MRS) and magnetic resonance imaging (MRI) with a two-point Dixon (2PD) method. Measurements were performed on a Philips 3 T system (Ingenuity TF PET/MR) with Q-Body coil, with seven participants measured on a Siemens Magnetom Skyra fit 3 T system (Siemens Healthcare, Erlangen, Germany) with Siemens Body 30 and 18 channel coils, and 32 channel Spine coil due to scanner replacement. For each individual, pre- and post-intervention scans were done on the same scanner.

For the Philips system, spectra were acquired with stimulated echo acquisition mode 1H MRS (repetition time (TR)/echo time (TE)/mixing time (TM) = 2000/11/17 ms, 4 averages) during 12 breath holds. The 3D T1-fast field echo sequence was acquired in the axial plane (TR/TE1/TE2 = 2.8/0.81/1.8 ms, flip angle 10°) with respiratory gating. For the Siemens system, point resolved spectroscopy 1H MRS was used (TR/TE = 4000/30 ms, 32 averages) with a respiratory navigator. A 3D gradient echo volumetric interpolated breath-hold examination (VIBE Dixon) sequence was acquired axially (TR/TE1/TE2 = 3.97/1.23/2.46 ms, flip angle 9°) with breath holds and controlled aliasing in parallel imaging results in higher acceleration (CAIPIRINHA) technique. LC Model (Version 6.3-0 C) quantified liver fat (spectrum type ‘liver-4′) using lipid signals at 1.6, 1.3, and 0.9 ppm. Signals were corrected for T2 decay and molar concentrations of 1H nuclei. LFC was defined as fat relative to total liver tissue weight. MRI images were analyzed with Carimas software (v2.10, Turku PET Centre, Turku, Finland). Four representative regions of interest were manually drawn on liver sections without main portal veins (left lateral and medial, right anterior and posterior), and volume correction was applied.

### Echocardiographic measurements

As previously described [39], two experienced clinical physiologists (M.S. and P.K) performed transthoracic echocardiographic measurements. Left ventricular (LV) wall thickness (posterior, septal), end-diastolic diameter (EDD), aortic root diameter and left atrial (LA) diameter were measured at the end of the diastole from parasternal long axis M-mode images. Subsequent, relative wall thickness (RWT = (LV posterior wall thickness * 2)/LVEDD) was calculated and LV mass and mass index (adjusted for body surface area) estimated according to the American Society of Echocardiography formula [40]. LV end-diastolic and end-systolic volumes (EDV and ESV), ejection fraction (EF) and stroke volume (SV = EDV-ESV), were measured from apical two- and four-chamber views using the Simpson biplane method. Cardiac output was calculated as stroke volume*hearth rate. Left atrial (LA) ESV index (adjusted for body surface area) was estimated from apical two- and four-chamber views using the biplane area-length method. Diastolic function was assessed by pulsed wave Doppler measurements of peak early (E) and peak late (atrial) (A) diastolic filling velocities and their ratio, E/A, and by tissue Doppler of lateral early diastolic mitral annular velocity (E’) with calculation of the E/E’ ratio. Global longitudinal strain (GLS) was measured by speckle tracking, averaging values from apical two-, three- and four-chamber views, at a frame rate above 50 frames per second. Image quality was assessed visually. In addition, GLS was assessed during the before described graded maximal exercise test on a recumbent cycle ergometer, starting at 25 W with load increments of 25 W every 3 min. GLS was measured after 2 min of each load level.

During transthoracic echocardiography single-lead electrocardiography was used to assess the heart rate and to synchronize image acquisition to the cardiac cycle. Transthoracic echocardiography was performed with Vivid E9 (GE Vingmed Ultrasound AS, Horton, Norway), and analyzed with Echopac plugin ViewPoint v6.12 (GE Healthcare, Solingen, Germany).

### Statistics

Baseline characteristics are reported separately for the intervention and control groups as absolute frequencies (%) for categorical variables and as mean (SD) for normally distributed variables and median (IQR) for non-normally distributed variables. The effects of the intervention, including group, time, sex and group*time, on the variables of interest, including Hb, Hct, RBC count, MCV, and MCH, were assessed using a linear mixed model for repeated measurements. The residual distribution was checked visually for normality. Akaike’s information criterion was used to choose between compound symmetry and unstructured covariance structure. Tukey-Kramer adjustment for multiple comparisons was used, and a two-sided significance level of *p* < 0.05 was used. Effects are reported as model-based means (95% CI). Correlations, at baseline and between changes, were calculated using Pearson’s correlation, unless the data deviated from normal distribution, in which case Spearman’s rank correlation was performed. Visual inspection was used to determine normal distribution. To adjust for BMI, partial correlation was performed. For variables requiring Spearman correlation, data was rank transformed prior to partial correlation analysis. SAS V.9.4 for Windows (SAS Institute Inc., Cary, NC, USA) and IBM SPSS Statistics for Windows, v30.0.0.0 (IBM Corp., Armonk, N.Y., USA) were used for statistical analyses.

## Results

### Baseline characteristics

A total of 64 participants (mean age: 58 years; 37 women) were included in the study, of whom 33 were allocated to the intervention group and 31 to the control group. One and three participants of the groups, respectively, discontinued the study. The flow of participants is shown in a study flow chart (Supplemental Figure 1). RBC parameters at baseline are presented in Table 1. Three participants had Hb levels below and two participants had Hb levels above the Finnish reference values but were still included as the study’s main aim was to compare changes in Hb levels over time. Baseline characteristics, including data on anthropometrics, PA, dietary intake, metabolic parameters, liver parameters, and echocardiographic parameters, are presented in Supplemental Table 1.

**Table 1.** Baseline characteristics of participants.

	Intervention	Control
n (%)	33 (52)	31 (48)
Sex, women, n (%)	20 (61)	17 (55)
Age, years	59.3 (6.0)	57.2 (7.5)
**Red blood cell parameters**		
RBC count,10^12^/L	4.64 (0.4)	4.57 (0.44)
Hb, g/L	141 (11)	139 (12)
Hct, %	41.7 (3.1)	41.0 (3.2)
MCV, fl	89.9 (4.2)	89.8 (3.1)
MCH, pg	30.4 (1.6)	30.5 (1.3)

RBC = red blood cell; Hb = Hemoglobin; Hct = Hematocrit; MVC = mean corpuscular volume; MCH = mean corpuscular hemoglobin.

Values are presented as mean (SD).

### Baseline correlations

Baseline cross-sectional correlations are represented in Supplemental Table 2. Correlations indicated that before the intervention individuals with lower Hb levels had lower total body weight (*r* = 0.27, *p* = 0.031) and waist circumference (*r* = 0.27 *p* = 0.032) as well as lower fat-free mass (*r* = 0.44, *p* < 0.001) and higher body fat percentage (r = -0.31, *p* = 0.013). Higher Hb levels correlated with less time spent standing (r=-0.35, *p* = 0.005). Whole body glucose uptake during HEC showed a negative correlation with Hb levels, while HOMA-IR showed a positive association (r_s _= -0.35, *p* = 0.004 and r_s _= 0.27, *p* = 0.031, respectively), suggesting that individuals with lower Hb levels had higher insulin sensitivity. Both resting O_2_ consumption and resting energy expenditure were positively correlated with Hb levels (*r* = 0.41, *p* < 0.001 and r_s _= 0.49, *p* < 0.001, respectively) and exercise-stimulated metabolic flexibility was negatively correlated with Hb levels (r = -0.34, *p* = 0.010).

The variables mentioned above that correlated with Hb levels were also statistically significantly correlated with RBC count. Hct showed similar patterns, except for non-significant correlations with body weight and exercise-stimulated metabolic flexibility (Supplemental Table 2). In addition, higher RBC count correlated with more sedentary time, higher fasting insulin levels and with lower HDL. MCV correlated negatively with weight, FFM, sedentary time, and resting O_2_ consumption and positively with body fat %, LPA, cholesterol and HDL. MCH correlated positively with LPA (Supplemental Table 2).

Dietary assessment showed that higher Hb levels correlated with higher energy intake in kcal/d (*r* = 0.28, *p* = 0.028) and with higher carbohydrate intake in g/d (*r* = 0.25, *p* = 0.043).

Among the liver parameters, higher LFC correlated with higher Hb levels (r_s _= 0.40, *p* = 0.011) and Hct. RBC count correlated negatively, and MCV positively with LGU. Higher RBC count and Hct correlated with higher GGT levels. MCH showed a negative correlation with EGP (Supplemental Table 2).

Echocardiographic measurements at baseline showed that higher Hb levels correlated with higher wall thicknesses, including septum (*r* = 0.34, *p* = 0.007), LV posterior wall (*r* = 0.42, *p* = 0.001), and relative wall thickness (*r* = 0.34, *p* = 0.007) and with higher LV mass, both as absolute mass (*r* = 0.32, *p* = 0.010) and indexed to body surface area (*r* = 0.29, *p* = 0.021), and a higher aortic root diameter (*r* = 0.34, *p* = 0.006). These variables also correlated significantly with RBC count and Hct. Higher Hb levels correlated with higher, thus worse, resting GLS (*r* = 0.27 *p* = 0.041), but this correlation was reversed during exercise at 50 W and 75 W intensity (r = -0.43 *p* = 0.004 and r = -0.40, *p* = 0.013, respectively). MCV showed a negative correlation with septum and LV posterior wall thickness, LV mass and aortic root diameter, and LVESV and MCH correlated positively with LVESV only (Supplemental Table 2).

### Intervention effects and changes over time

#### Sedentary behavior and physical activity

As previously reported [25], during the 6-month intervention period, the intervention group reduced daily sedentary time by 40 (95% CI: 17, 65) min/day and increased MVPA by 20 (95% CI: 11, 28) min/day, compared with nonsignificant changes in the control group. LPA and daily steps increased in both groups, with greater changes in steps in the intervention group. However, the reduction in SB varied considerably among participants [25].

#### Hemoglobin and red blood cell parameters

**Figure 1.**
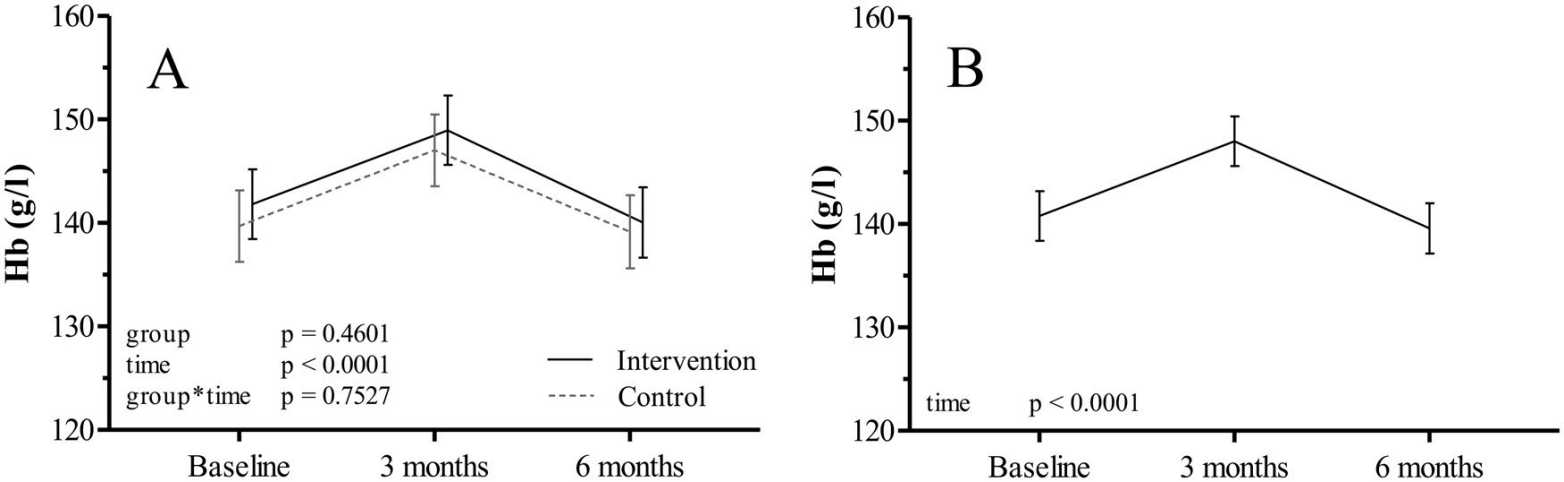
Hb level changes during the six-month intervention period. A: Comparison of the changes in Hb levels (g/l) between intervention (*n* = 33) and control (*n* = 31) group B: Changes in Hb levels (g/l) of all participants over time.

Over the six-month period, there were no differences in Hb levels between the intervention and the control group (group*time *p* > 0.05) (Figure 1A and Supplemental Table 3). Among all participants, Hb levels increased on average by 7.24 g/l (95% CI: 5.59, 8.89) from baseline to 3 months (time *p* < 0.001), but returned close to baseline by 6 months, resulting in a non-significant mean overall change of −1.2 g/l (95% CI: −2.90, 0.50) (Figure 1B). As individuals were continuously enrolled over more than two years (Supplemental Figure 2), seasonal effects or systemic issues during blood collection are unlikely to have contributed to the changes in Hb levels.

Consistent with the data on Hb levels, there were no differences between groups in RBC count, Hct, MCV and MCH. (Supplemental Table 3).

### Correlations between changes from baseline to 3 months

Correlation coefficients for available parameters between the changes over three months are depicted in Supplemental Table 4. An increase in fasting blood glucose levels correlated with an increase in Hb levels (*r* = 0.28, *p* = 0.031), otherwise no other statistically significant correlations with changes in Hb levels, Hct, or RBC count were found. However, decreased body fat % and fat mass and increased FFM correlated with an increase in MCV. MCH showed a similar pattern, with a negative correlation with body fat % and a positive correlation with FFM.

### Correlations between changes from baseline to 6 months

#### Anthropometrics

At six months, reductions in body weight and BMI correlated with decreases in Hct (*r* = 0.31, *p* = 0.021 and *r* = 0.32, *p* = 0.014, respectively). Additionally, changes in BMI and RBC count correlated positively (*r* = 0.28, *p* = 0.038). The correlations between changes in anthropometric measures and MCV or MCH observed at three months were non-significant after six months. All correlation coefficients are shown in Table 2.

**Table 2.** Correlation coefficients between changes in RBC parameters and anthropometric variables after six-month intervention (*n* = 57).

Anthropometrics	ΔHb, g/L	ΔHct, %	ΔRBC count,10^12^/l	ΔMCV, fl	ΔMCH, pg
ΔWeight, kg	0.25	0.31*	0.26	0.01	−0.11
ΔBMI, kg/m^2^	0.26	0.32*	0.28*	0.01	−0.13
ΔWaist circumference, cm ^†^	−0.06	−0.01	−0.07	−0.01	−0.02
ΔBody fat-%	0.03	0.02	0.09	−0.23	−0.04
ΔFat mass, kg	0.11	0.12	0.16	−0.21	−0.08
ΔFat-free mass, kg ^rs^	0.18	0.17	0.11	0.25	0.00

Red indicates positive and blue negative correlation. Pearson’s correlation was performed unless data deviated from normality, in which case Spearman’s rank correlation (marked with = rs) was used. Bold values indicate statistical significance: * *= p* < 0.05. ^†^=*n* = 52.

#### Physical activity

**Figure 2.**
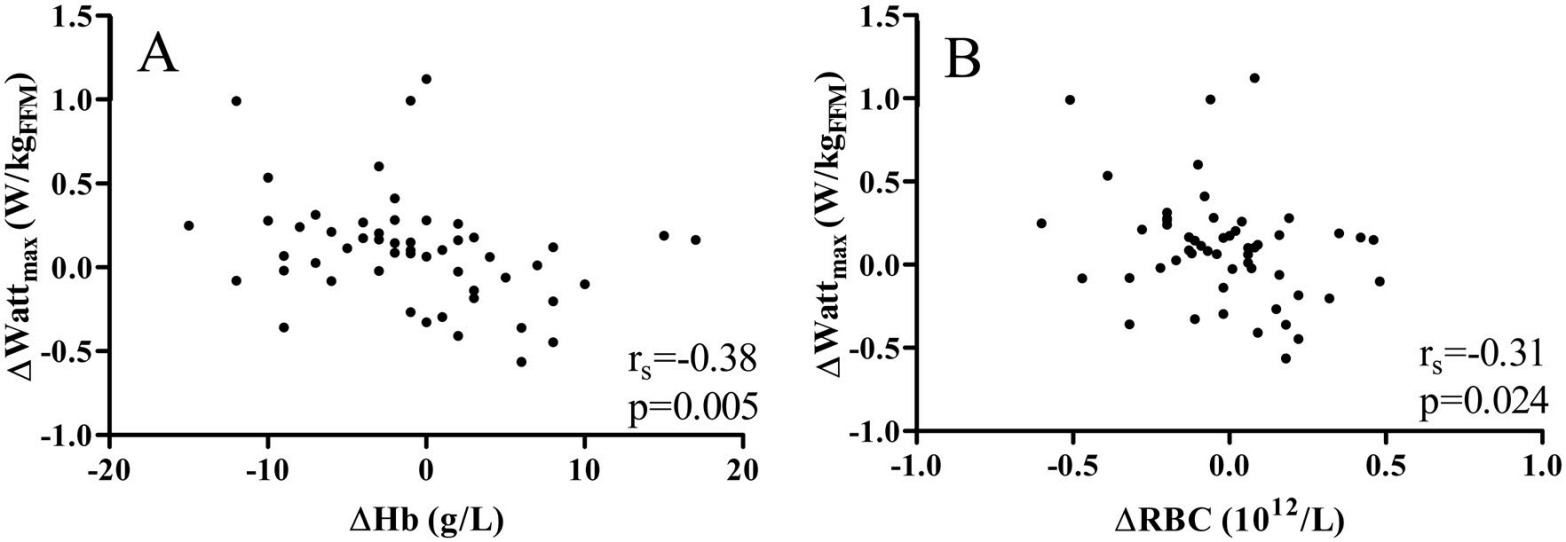
Correlations between changes after the six-month intervention in maximal power output (W_max_/kg_FFM_) and A: Hb levels and B: RBC count.

No statistically significant correlations were seen between changes in PA or SB measures and RBC-related outcomes, except for a positive correlation between change in step count and change in MCH. However, increased maximal power output correlated with decreased Hb levels (W_max_/kg: r_s _= -0.29, *p* = 0.033 and W_max_/kg_FFM_: r_s _= -0.38, *p* = 0.005) and decreased RBC count (W_max_/kg_FFM_: r_s _= -0.31, *p* = 0.024) (Figure 2). All correlation coefficients are shown in Supplemental Table 5.

#### Dietary intake

Changes in total energy and macronutrient intake showed no correlation with changes in Hb levels or other RBC parameters. Positive correlations were detected for changes in polyunsaturated fatty acids intake in g/d with changes in Hct and RBC count, and additionally in % of total energy intake with changes in Hb levels. Changes in monounsaturated fatty acid intake correlated positively with changes in RBC count and negatively with changes in MCH. (Supplemental Table 6).

#### Insulin sensitivity and blood lipids

**Figure 3.**
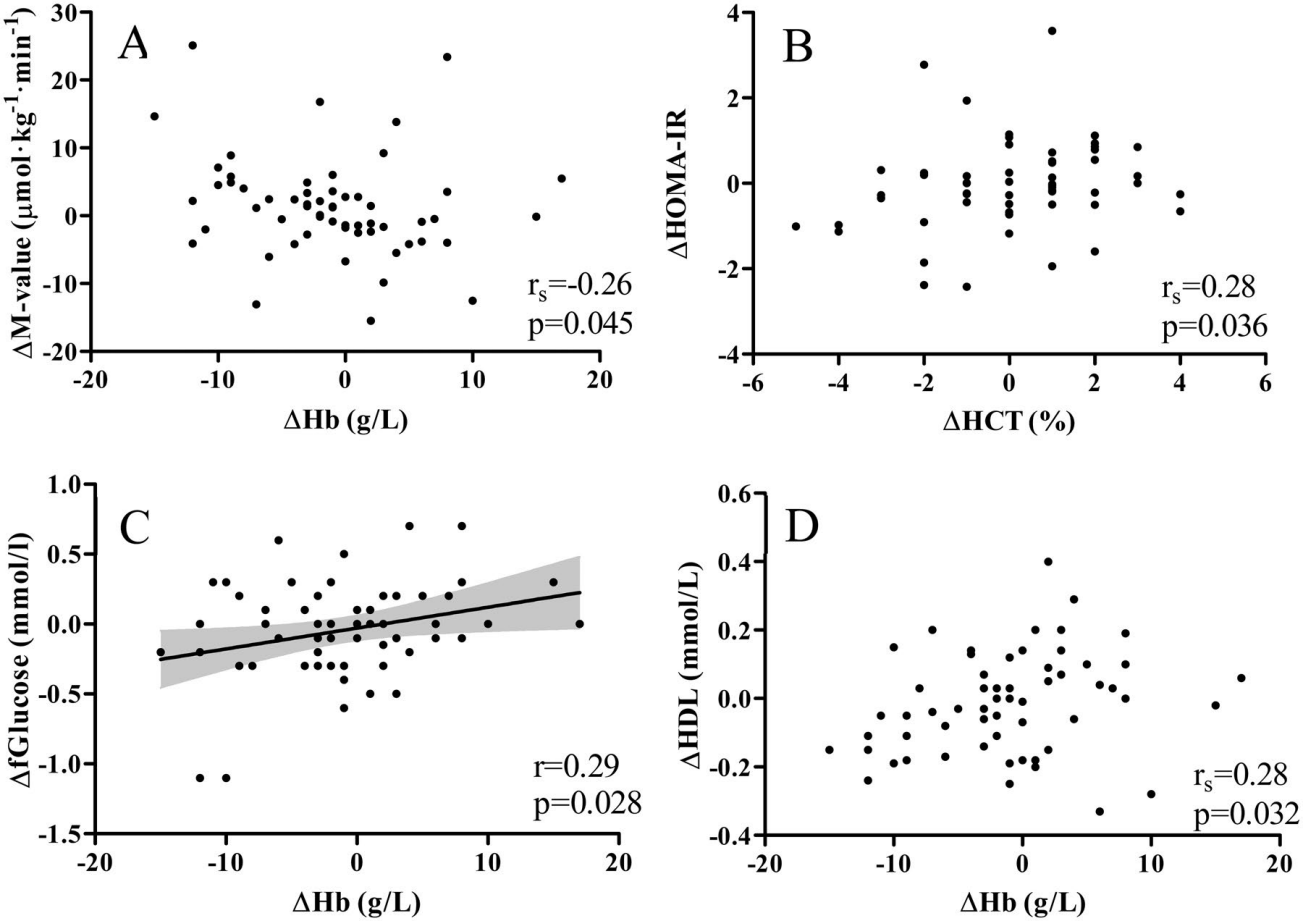
Correlations between changes after six months of A: Hb levels and M-value, B: Hct and HOMA-IR, C: Hb levels and fasting blood glucose levels, D: Hb levels and HDL.

Changes in the M-values, i.e. whole-body insulin sensitivity under insulin-clamp condition, correlated negatively with the changes in Hb levels (r_s _= -0.26, *p* = 0.045) (Figure 3A) suggesting that increased insulin sensitivity is associated with decreased Hb levels. In addition, decreased HOMA-IR correlated with decreased Hct (r_s _= 0.28, *p* = 0.036) (Figure 3B) and decreases in fasting blood glucose levels correlated with decreases in Hb levels (Figure 3C), Hct and RBC count (*r* = 0.29, *p* = 0.028, *r* = 0.28, *p* = 0.032, and *r* = 0.29, *p* = 0.026, respectively). Decreased HbA1c correlated with an increase in MCV (Supplemental Table 7). No significant correlations were observed between changes in Hb levels and other RBC parameters and changes in O_2_ consumption and energy expenditure at rest, or changes in insulin- and exercise-stimulated metabolic flexibility. Among blood lipid levels, increases in HDL correlated with increases in Hb levels (Figure 3D), Hct and RBC count (r_s _= 0.28, *p* = 0.032, r_s _= 0.28, *p* = 0.037, and r_s _= 0.28, *p* = 0.036, respectively) (Supplemental Table 7).

#### Liver parameters

Among the liver parameters, shown in Supplemental Table 8, the positive correlation between changes in LFC and RBC count (r_s _= 0.36, *p* = 0.039) was the only statistically significant finding as the changes in LGU, EGP and liver enzymes, including AST, ALT, and GGT, were not correlated with changes in RBC parameters.

#### Echocardiographic parameters

In contrast to baseline, no correlations were observed between changes in echocardiographic variables and changes in Hb levels, Hct, or RBC count over the six months. Increases in MCV correlated with decreased LV posterior wall thickness and relative wall thickness and with increased LVEDD and LVESV. Also, changes in MCH correlated negatively with changes in relative wall thickness and positively with LVEDD (Supplemental Table 9).

#### BMI adjustment

In an additional analysis (Supplemental Table 10), the correlations between changes observed during the intervention period were adjusted for changes in BMI, which turned most of the correlations statistically non-significant. However, correlations that appeared to be independent of BMI changes included those between changes in step count and MCH, changes in maximal power output per FFM and Hb levels, and changes in HDL and Hb levels, Hct, and RBC count. Furthermore, changes in monounsaturated fatty acid intake (in g/d or % of total energy intake) remained correlated with changes in MCH and RBC count after adjusting for BMI changes, as did correlations between changes in Hb or MCV and changes in echocardiographic parameters. Also, the correlation between changes in HbA1c and MCV remained strong regardless of changes in BMI. In addition, after adjustment for BMI changes, the correlations of changes in MCV with changes in FFM and monounsaturated fatty acid intake turned significant.

## Discussion

In the present study we aimed to test the hypothesis that changes in Hb levels, body adiposity, and associated cardiometabolic derangements would correlate with each other if they were causally related. While there were numerous significant correlations between Hb levels and cardiometabolic outcomes assessed cross-sectionally at baseline, many of these associations did not persist when changes between these variables were investigated. Nevertheless, over the time of the intervention, reductions in Hb levels correlated significantly with improved whole-body insulin sensitivity during the gold-standard insulin clamp measurement. Interestingly, decreases in Hb levels were also correlated favorably with increased exercise capacity, and decreased blood glucose levels. Similar trends were found for RBC count and Hct. Most correlations were, however, not independent of changes in BMI, suggesting that adiposity plays a crucial role within this association.

No significant changes in Hb levels or other RBC parameters were detected by the six-month SB reduction intervention in these individuals with metabolic syndrome and no differences were found between the intervention and control groups. As the SB reduction was not designed to induce substantial Hb level changes, this may have limited the ability to detect stronger correlations over time. However, modest within-individual changes were observed and their correlations with changes in glucose metabolism and exercise capacity indicate that even small changes within physiological ranges may be clinically relevant in individuals with metabolic syndrome. Although we cannot provide mechanistic evidence for a causal inference, some of our findings align with recent evidence that suggests a potential causal role of Hb levels on cardiometabolic outcomes, mediated by tissue oxygenation and the hypoxia-inducible factor (HIF) pathway [2]. This recent comprehensive study [2] proposed that a mild hypoxic state due to lower Hb levels may lead to the upregulation of HIF target gene expression, causing changes in energy metabolism, such as reduced oxidative phosphorylation and increased glycolysis and glucose uptake [41]. Those alterations are associated with benefits for metabolic health. Although Mendelian randomization analyses showed mixed results [2,42], gene expression data and Hb level manipulation in animal models support a potential causal relationship [2]. In addition, several preclinical studies have shown that the inhibition or knockout of HIF prolyl 4-hydroxylases (HIF-P4Hs), in particular its isoenzyme 2 [43], has beneficial effects on metabolic outcomes in mice [43–47]. In humans, lipid profiles improved in patients receiving a pan-HIF-P4H inhibitor [48], which has been approved to treat anemia in patients with chronic kidney disease [49]. Improved glucose metabolism and a lower prevalence of obesity have been observed in individuals residing at higher altitudes, further suggesting a potential beneficial effect of hypoxia [50–53].

### Hb levels and metabolic changes

One of our main findings was the relationship between decreased Hb levels and improved insulin sensitivity. Our baseline data revealed significant correlations between Hb levels and metabolic parameters, similar to previous cross-sectional studies [2–6]. While in our findings, insulin and blood glucose levels were not significantly correlated at baseline, HOMA-IR score and whole-body insulin sensitivity showed that low Hb levels associated with better insulin sensitivity and importantly also over time, changes in these parameters correlated. In combination with the correlation between decreased blood glucose levels and decreased Hb levels, RBC count, and Hct, these correlations suggest that decreases in Hb levels may contribute to improved insulin sensitivity, or conversely, that improvements in metabolic health may lead to reduced Hb levels.

### Hb levels and physical activity

Interestingly, decreases in Hb levels were correlated with increased power output during cycle ergometer tests, scaled to total body weight and FFM. This is in contrast with the usual link between higher Hb levels and better exercise performance [54], suggesting that in individuals with metabolic syndrome, the systemic metabolic improvements associated with decreased Hb levels, may outweigh its classical role in oxygen transport capacity. Notably, the changes in VO_2_max did not correlate significantly with the changes in Hb levels, suggesting that increased power output may be driven by adaptations other than improved maximal aerobic capacity.

Prolonged sedentary time has been associated with adverse effects on cardiometabolic health in previous studies [33,55,56]. However, the effect of SB independent of PA has also been questioned, emphasizing that PA, including innovative approaches [57,58], is necessary for improving cardiometabolic outcomes [20,59,60]. While higher SB was correlated with higher Hb levels at baseline, no correlation was detected over time. The six-month reduction in SB did not have a significant impact on Hb levels, suggesting that the magnitude of SB reduction was likely insufficient and baseline correlations may reflect long-term behavior, not modified within six months. Clinically, this suggests that for individuals at risk, changes in Hb levels, and potentially related metabolic benefits, may require larger and sustained reductions in sedentary time combined with increased PA.

### Hb levels and anthropometrics

Similar to previous cross-sectional reports [3,4], at baseline, higher Hb levels were correlated with higher body weight and waist circumference, though not significantly with BMI. In a previous study [2], this correlation was attributed to higher fat and lower lean mass, conflicting with our data, in which higher Hb levels were associated with lower body fat percentage and higher FFM. Over time, however, decreasing RBC count and Hct levels were correlated with decreases in BMI in the present study. Together with the fact that many of the longitudinal correlations were attenuated after BMI adjustment, these findings suggest that changes in body composition may mediate both Hb level changes and metabolic improvements.

### Hb levels and liver health

Previously, high Hb levels have been linked to an increased risk of metabolic dysfunction-associated steatotic liver disease [61]. In addition, iron metabolism is closely linked to liver function, with elevated iron levels adversely affecting hepatic health [62]. In our data, the positive correlation between LFC and Hb levels at baseline, and between changes in LFC and RBC over six months, suggests a potential link with hepatic steatosis. However, changes in liver insulin sensitivity and liver enzyme levels showed no correlation with changes in RBC parameters over time. As concluded in a previous analysis using the same dataset (34), sedentary reduction alone may be insufficient to significantly change liver parameters, potentially also limiting correlations with Hb levels.

### Hb levels and cardiac function

Two recent cross-sectional studies [11,13] reported an association between higher Hb levels and increased GLS values, indicating impaired cardiac function, in both healthy individuals [11] and individuals with metabolic syndrome [13]. Our data agreed with these findings and also showed a correlation between higher Hb levels and worse cardiac function, as indicated by higher GLS, wall thicknesses, and LV mass. Additionally, we found a correlation with aortic root diameter. Moreover, we also examined GLS values during exercise. Surprisingly, the correlation changed: higher Hb levels were significantly associated with lower GLS values, indicating better cardiac function, at light intensity (50 W and 75 W). This may be explained by the increased importance of the benefits of increased oxygen-carrying capacity during exercise. Over time, changes in echocardiographic parameters only correlated with changes in MCV and MCH, not with Hb levels, RBC count, or Hct.

### Clinical implications

Hb levels are routinely measured and usually evaluated in the context of anemia. Our findings suggest that Hb levels within the reference range may also reflect cardiometabolic health. Since metabolic syndrome is highly prevalent and associated with an increased risk of cardiovascular events and mortality, proper risk stratification and monitoring are essential. Further studies are needed to assess if including Hb levels improves risk prediction compared to existing models. However, our findings suggest that even subtle changes in Hb levels may reflect changes in metabolic health and could help track responses to lifestyle modifications. Hb levels at the high end of the reference range could prompt a closer assessment of cardiometabolic risk factors. Our study also showed that SB reduction alone is not sufficient to change Hb levels and highlights the role of body adiposity. Finally, while we could not establish causality in this study, the observed correlations align with evidence suggesting that Hb levels and tissue oxygenation may modulate metabolic pathways. Future clinical interventions could explore whether interventions that modulate Hb levels or tissue oxygenation, such as altitude or hypoxic exposure, might offer additional metabolic benefits.

### Strengths and limitations

The strengths of our study include its randomized controlled intervention design with longitudinal follow-up, which allows for analysis over time compared to previous cross-sectional data. Additionally, our study included a comprehensive assessment of multiple cardiometabolic parameters, including HEC for insulin sensitivity, MRS for liver fat content, and GLS during exercise. In addition, the study provided continuous accelerometer data over six months, allowing objective measurement of PA behavior. Furthermore, the study population of individuals with metabolic syndrome is highly relevant in today’s clinical context. It represents a population at high cardiovascular risk which is enhancing the translational potential of our findings. The strength of our study does not lie in one single observation but in its broad approach of evaluating hematological, cardiometabolic, and behavioral outcomes allowing the detection of trends and the generation of hypotheses.

Limitations include the relatively small sample size, as the study was powered for the primary outcome of whole-body insulin sensitivity rather than the secondary analysis presented here. The ability to detect significant correlations may have been further limited by the modest effect size of the SB reduction. Six-months may have been too short to detect adaptations in Hb levels, RBC parameters and their effect on cardiometabolic outcomes. To address this issue, larger studies that are powered to detect small longitudinal associations or RCTs aimed directly at Hb level changes, such as altitude exposure, would be valuable. Further, no correction for multiple testing was applied. This decision was made deliberately to preserve sensitivity for detecting potential trends, however, findings should be seen as hypothesis-generating and need to be further evaluated. Hb concentration measurements may be influenced by plasma volume changes, which can mask changes in total Hb mass, which cannot be determined by Hb concentration measurement. It might be that the total blood volume does not increase linearly with the BMI and thus total Hb mass measurements would provide more insights [63]. We did not assess further RBC- and iron-related factors or inflammatory parameters which could help to understand underlying pathways even further.

## Conclusions

In individuals with metabolic syndrome, a six-month SB reduction intervention did not significantly impact Hb levels or other RBC parameters. While baseline correlations supported an association between higher Hb levels and poorer cardiometabolic health, changes during the intervention did not consistently correlate. Nevertheless, reduced Hb levels during the intervention correlated with improved whole-body insulin sensitivity and maximal aerobic fitness, with body adiposity being a key factor in this relationship. Although we could not establish causality, these longitudinal results suggest that high Hb levels may be both a marker and a driver of metabolic dysfunction, and that subtle changes in Hb levels could be clinically relevant. Given the high prevalence of metabolic syndrome and its associated risks, Hb level changes may be useful for risk stratification and monitoring of affected individuals. However, further mechanistic and intervention studies with more pronounced effects and total Hb mass determinations are needed to further assess this potential relationship and explore diagnostic and therapeutic applications.

## Supplementary Material

Supplemental file.docx

## Data Availability

Data are available from the corresponding author upon reasonable request.

## References

[1] Kairisto V, Grönroos P, Loikkanen M, et al. New Finnish reference limits for the basic blood count. Suom Laakaril. 2003;58:5147–5153.

[2] Auvinen J, Tapio J, Karhunen V, et al. Systematic evaluation of the association between hemoglobin levels and metabolic profile implicates beneficial effects of hypoxia. Sci Adv. 2021;7(29):eabi4822. doi: 10.1126/sciadv.abi4822. 34261659 PMC8279517

[3] Koivula T, Lempiäinen S, Laine S, et al. Cross-sectional associations of body adiposity, sedentary behavior, and physical activity with hemoglobin and white blood cell count. Int J Environ Res Public Health. 2022;19(21):14347. doi: 10.3390/ijerph192114347. 36361221 PMC9657926

[4] Hämäläinen P, Saltevo J, Kautiainen H, et al. Erythropoietin, ferritin, haptoglobin, hemoglobin and transferrin receptor in metabolic syndrome: a case control study. Cardiovasc Diabetol. 2012;11(1):116. doi: 10.1186/1475-2840-11-116. 23016887 PMC3471017

[5] Facchini FS, Carantoni M, Jeppesen J, et al. Hematocrit and hemoglobin are independently related to insulin resistance and compensatory hyperinsulinemia in healthy, non-obese men and women. Metabolism. 1998;47(7):831–835. doi: 10.1016/s0026-0495(98)90121-4. 9667230

[6] Kawamoto R, Tabara Y, Kohara K, et al. Hematological parameters are associated with metabolic syndrome in Japanese community-dwelling persons. Endocrine. 2013;43(2):334–341. doi: 10.1007/s12020-012-9662-7. 23307027

[7] Sakko S, Karpale M, Tapio J, et al. Hemoglobin levels are associated with retinal vascular caliber in a middle-aged birth cohort. Sci Rep. 2024;14(1):9092. doi: 10.1038/s41598-024-59688-y. 38643302 PMC11032340

[8] Tapiola A, Tapio J, Vähänikkilä H, et al. Higher haemoglobin levels are associated with impaired periodontal status. J Clin Periodontol. 2024;51(9):1168–1177. doi: 10.1111/jcpe.14030. 38872488

[9] Li Y, Wang F, Huang X, et al. First-trimester hemoglobin, haptoglobin genotype, and risk of gestational diabetes mellitus in a retrospective study among Chinese pregnant women. Nutr Diabetes. 2024;14(1):48. doi: 10.1038/s41387-024-00309-y. 38951151 PMC11217379

[10] Sissala N, Mustaniemi S, Kajantie E, et al. Higher hemoglobin levels are an independent risk factor for gestational diabetes. Sci Rep. 2022;12(1):1686. doi: 10.1038/s41598-022-05801-y. 35102239 PMC8803843

[11] Tapio J, Grönlund T, Kaikkonen K, et al. Haemoglobin levels are associated with echocardiographic measures in a Finnish midlife population. Ann Med. 2024;56(1):2425061. doi: 10.1080/07853890.2024.2425061. 39624964 PMC11616746

[12] Tapio J, Kiviniemi AM, Perkiömäki J, et al. Lower hemoglobin levels associate with higher baroreflex sensitivity and heart rate variability. Am J Physiol Heart Circ Physiol. 2023;325(4):H629–H634. doi: 10.1152/ajpheart.00415.2023. 37566112 PMC10659262

[13] Li X, Yu S-Q, Yang Z-G, et al. Higher hemoglobin levels are associated with impaired left ventricular global strains in metabolic syndrome: a 3.0 T CMR feature tracking study. Cardiovasc Diabetol. 2025;24(1):123. doi: 10.1186/s12933-025-02664-1. 40097959 PMC11916957

[14] Wang G, Mao W, Zhang Y, et al. Multiomics and systematic analyses reveal the roles of hemoglobin and the HIF-1 pathway in polycystic ovary syndrome. Adv Sci (Weinh). 2025;12(14):e2411679. doi: 10.1002/advs.202411679. 39950870 PMC11984896

[15] Tapio J, Vähänikkilä H, Kesäniemi YA, et al. Higher hemoglobin levels are an independent risk factor for adverse metabolism and higher mortality in a 20-year follow-up. Sci Rep. 2021;11(1):19936. doi: 10.1038/s41598-021-99217-9. 34620927 PMC8497471

[16] Grundy SM, Cleeman JI, Daniels SR, et al. Diagnosis and management of the metabolic syndrome: an American Heart Association/National Heart, Lung, and Blood Institute Scientific Statement. Circulation. 2005;112(17):2735–2752. doi: 10.1161/CIRCULATIONAHA.105.169404. 16157765

[17] Hashimoto Y, Tanaka M, Kimura T, et al. Hemoglobin concentration and incident metabolic syndrome: a population-based large-scale cohort study. Endocrine. 2015;50(2):390–396. doi: 10.1007/s12020-015-0587-9. 25863486

[18] Choudhary MK, Bouquin H, Hytönen J, et al. Blood haemoglobin concentration is directly and independently related with pulse wave velocity, a measure of large artery stiffness. J Clin Med. 2023;12(24):7623. doi: 10.3390/jcm12247623. 38137695 PMC10743951

[19] Garthwaite T, Sjöros T, Koivumäki M, et al. Standing is associated with insulin sensitivity in adults with metabolic syndrome. J Sci Med Sport. 2021;24(12):1255–1260. J Sci Med Sport. 2022;25(6):541. doi: 10.1016/j.jsams.2021.08.009. 34489177

[20] Heinonen I. Clinical and physiological advances in sedentary behavior research. Front Physiol. 2024;15:1348122. doi: 10.3389/fphys.2024.1348122. 38550258 PMC10973114

[21] Norha J, Sjöros T, Garthwaite T, et al. Effects of reduced sedentary time on resting, exercise and post-exercise blood pressure in inactive adults with metabolic syndrome - a six-month exploratory RCT. J Hum Hypertens. 2024;38(4):314–321. doi: 10.1038/s41371-024-00894-6. 38267651 PMC11001575

[22] Norha J, Sjöros T, Garthwaite T, et al. Effects of reducing sedentary behavior on cardiorespiratory fitness in adults with metabolic syndrome: a 6-month RCT. Scand J Med Sci Sports. 2023;33(8):1452–1461. doi: 10.1111/sms.14371. 37073456

[23] Norha J, Sjöros T, Garthwaite T, et al. Effects of reducing sedentary behaviour on back pain, paraspinal muscle insulin sensitivity and muscle fat fraction and their associations: a secondary analysis of a 6-month randomised controlled trial. BMJ Open. 2024;14(9):e084305. doi: 10.1136/bmjopen-2024-084305. PMC11440184 39343453

[24] Sjöros T, Laine S, Garthwaite T, et al. The effects of a 6-month intervention aimed to reduce sedentary time on skeletal muscle insulin sensitivity: a randomized controlled trial. Am J Physiol Endocrinol Metab. 2023;325(2):E152–e62. doi: 10.1152/ajpendo.00018.2023. 37378623

[25] Sjöros T, Laine S, Garthwaite T, et al. Reducing sedentary time and whole-body insulin sensitivity in metabolic syndrome: a 6-month randomized controlled trial. Med Sci Sports Exerc. 2023;55(3):342–353. doi: 10.1249/MSS.0000000000003054. 36251378 PMC9924963

[26] Ylinen VP, Sjöros T, Laine S, et al. Sedentary behavior reduction and blood lipids in adults with metabolic syndrome: a 6-month randomized controlled trial. Sci Rep. 2024;14(1):24241. doi: 10.1038/s41598-024-75579-8. 39414998 PMC11484901

[27] Garthwaite T, Sjöros T, Laine S, et al. Effects of reduced sedentary time on cardiometabolic health in adults with metabolic syndrome: a three-month randomized controlled trial. J Sci Med Sport. 2022;25(7):579–585. doi: 10.1016/j.jsams.2022.04.002. 35487860

[28] Alberti KGMM, Eckel RH, Grundy SM, et al. Harmonizing the metabolic syndrome: a joint interim statement of the International Diabetes Federation Task Force on Epidemiology and Prevention; National Heart, Lung, and Blood Institute; American Heart Association; World Heart Federation; International Atherosclerosis Society; and International Association for. The Study of Obesity. Circulation. 2009;120(16):1640–1645. doi: 10.1161/CIRCULATIONAHA.109.192644. 19805654

[29] Sjöros T, Vähä-Ypyä H, Laine S, et al. Both sedentary time and physical activity are associated with cardiometabolic health in overweight adults in a 1 month accelerometer measurement. Sci Rep. 2020;10(1):20578. doi: 10.1038/s41598-020-77637-3. 33239818 PMC7688927

[30] Laine S, Sjöros T, Vähä-Ypyä H, et al. Body adiposity, but not elements of objectively measured sedentary behavior or physical activity, is associated with circulating liver enzymes in adults with overweight and obesity. Front Endocrinol (Lausanne). 2021;12:655756. doi: 10.3389/fendo.2021.655756. 33959099 PMC8095079

[31] Vähä-Ypyä H, Vasankari T, Husu P, et al. Validation of Cut-points for evaluating the intensity of physical activity with accelerometry-based mean amplitude deviation (MAD). PLoS One. 2015;10(8):e0134813. doi: 10.1371/journal.pone.0134813. 26292225 PMC4546343

[32] Vähä-Ypyä H, Husu P, Suni J, et al. Reliable recognition of lying, sitting, and standing with a hip-worn accelerometer. Scand J Med Sci Sports. 2018;28(3):1092–1102. doi: 10.1111/sms.13017. 29144567

[33] Garthwaite T, Sjöros T, Laine S, et al. Sedentary time associates detrimentally and physical activity beneficially with metabolic flexibility in adults with metabolic syndrome. Am J Physiol Endocrinol Metab. 2024;326(4):E503–e14. doi: 10.1152/ajpendo.00338.2023. 38416072 PMC11194051

[34] Laine S, Sjöros T, Garthwaite T, et al. Effects of reducing sedentary behavior on liver insulin sensitivity, liver fat content, and liver enzyme levels: a six-month randomized controlled trial. Am J Physiol Endocrinol Metab. 2025;328(6):E756–E771. (doi: 10.1152/ajpendo.00446.2024. 40244864

[35] Laine S, Sjöros T, Garthwaite T, et al. Daily standing time, dietary fiber, and intake of unsaturated fatty acids are beneficially associated with hepatic insulin sensitivity in adults with metabolic syndrome. Front Endocrinol (Lausanne). 2024;15:1272886. doi: 10.3389/fendo.2024.1272886. 38989003 PMC11233550

[36] Laine S, Sjöros T, Garthwaite T, et al. Relationship between liver fat content and lifestyle factors in adults with metabolic syndrome. Sci Rep. 2022;12(1):17428. doi: 10.1038/s41598-022-22361-3. 36261605 PMC9581946

[37] Hamacher K, Coenen HH, Stöcklin G. Efficient stereospecific synthesis of no-carrier-added 2-[18F]-fluoro-2-deoxy-D-glucose using aminopolyether supported nucleophilic substitution. J Nucl Med. 1986;27(2):235–238. doi: 10.2967/jnumed.120.250191. 3712040

[38] Patlak CS, Blasberg RG. Graphical evaluation of blood-to-brain transfer constants from multiple-time uptake data. Generalizations. J Cereb Blood Flow Metab. 1985;5(4):584–590. doi: 10.1038/jcbfm.1985.87. 4055928

[39] Norha J, Saarenhovi M, Kallio P, et al. Effects of reducing sedentary behaviour on cardiac structure and function at rest and during exercise: a six-month randomised controlled Trial. CJC Open. 2026;8(1):69–81. doi: 10.1016/j.cjco.2025.09.005. 41732582 PMC12925755

[40] Lang RM, Badano LP, Mor-Avi V, et al. Recommendations for cardiac chamber quantification by echocardiography in adults: an update from the American Society of Echocardiography and the European Association of Cardiovascular Imaging. J Am Soc Echocardiogr. 2015;28(1):1–39.e14. doi: 10.1016/j.echo.2014.10.003. 25559473

[41] Kierans SJ, Taylor CT. Regulation of glycolysis by the hypoxia-inducible factor (HIF): implications for cellular physiology. J Physiol. 2021;599(1):23–37. doi: 10.1113/JP280572. 33006160

[42] Liu Y, Clarke R, Bennett DA, et al. Iron status and risk of heart disease, stroke, and diabetes: a mendelian randomization study in European adults. J Am Heart Assoc. 2024;13(6):e031732. doi: 10.1161/JAHA.123.031732. 38497484 PMC11010009

[43] Tapio J, Halmetoja R, Dimova EY, et al. Contribution of HIF-P4H isoenzyme inhibition to metabolism indicates major beneficial effects being conveyed by HIF-P4H-2 antagonism. J Biol Chem. 2022;298(8):102222. doi: 10.1016/j.jbc.2022.102222. 35787374 PMC9352911

[44] Laitakari A, Huttunen R, Kuvaja P, et al. Systemic long-term inactivation of hypoxia-inducible factor prolyl 4-hydroxylase 2 ameliorates aging-induced changes in mice without affecting their life span. Faseb J. 2020;34(4):5590–5609. doi: 10.1096/fj.201902331R. 32100354

[45] Laitakari A, Tapio J, Mäkelä KA, et al. HIF-P4H-2 inhibition enhances intestinal fructose metabolism and induces thermogenesis protecting against NAFLD. J Mol Med (Berl). 2020;98(5):719–731. doi: 10.1007/s00109-020-01903-0. 32296880 PMC7220983

[46] Rahtu-Korpela L, Karsikas S, Hörkkö S, et al. HIF prolyl 4-hydroxylase-2 inhibition improves glucose and lipid metabolism and protects against obesity and metabolic dysfunction. Diabetes. 2014;63(10):3324–3333. doi: 10.2337/db14-0472. 24789921

[47] Matsuura H, Ichiki T, Inoue E, et al. Prolyl hydroxylase domain protein 2 plays a critical role in diet-induced obesity and glucose intolerance. Circulation. 2013;127(21):2078–2087. doi: 10.1161/CIRCULATIONAHA.113.001742. 23630130

[48] Chen N, Hao C, Peng X, et al. Roxadustat for anemia in patients with kidney disease not receiving dialysis. N Engl J Med. 2019;381(11):1001–1010. doi: 10.1056/NEJMoa1813599. 31340089

[49] Dhillon S. Roxadustat: first global approval. Drugs. 2019;79(5):563–572. doi: 10.1007/s40265-019-01077-1. 30805897

[50] Voss JD, Allison DB, Webber BJ, et al. Lower obesity rate during residence at high altitude among a military population with frequent migration: a quasi experimental model for investigating spatial causation. PLoS One. 2014;9(4):e93493. doi: 10.1371/journal.pone.0093493. 24740173 PMC3989193

[51] Woolcott OO, Ader M, Bergman RN. Glucose homeostasis during short-term and prolonged exposure to high altitudes. Endocr Rev. 2015;36(2):149–173. doi: 10.1210/er.2014-1063. 25675133 PMC4399271

[52] Heinonen IH, Boushel R, Kalliokoski KK. The circulatory and metabolic responses to hypoxia in humans - with special reference to adipose tissue physiology and obesity. Front Endocrinol (Lausanne). 2016;7:116. doi: 10.3389/fendo.2016.00116. 27621722 PMC5002918

[53] Voss JD, Masuoka P, Webber BJ, et al. Association of elevation, urbanization and ambient temperature with obesity prevalence in the United States. Int J Obes (Lond). 2013;37(10):1407–1412. doi: 10.1038/ijo.2013.5. 23357956

[54] Webb KL, Gorman EK, Morkeberg OH, et al. The relationship between hemoglobin and V˙O2max: a systematic review and meta-analysis. PLoS One. 2023;18(10):e0292835. doi: 10.1371/journal.pone.0292835. 37824583 PMC10569622

[55] Healy GN, Matthews CE, Dunstan DW, et al. Sedentary time and cardio-metabolic biomarkers in US adults: NHANES 2003-06. Eur Heart J. 2011;32(5):590–597. doi: 10.1093/eurheartj/ehq451. 21224291 PMC3634159

[56] Lee IM, Shiroma EJ, Lobelo F, et al. Effect of physical inactivity on major non-communicable diseases worldwide: an analysis of burden of disease and life expectancy. Lancet. 2012;380(9838):219–229. doi: 10.1016/S0140-6736(12)61031-9. 22818936 PMC3645500

[57] Ali Ismail AM, El-Moatasem AM, El-Moatasem AM. Effect of baduanjin exercise on salivary inflammatory and oxidative markers in the elderly with metabolic syndrome and periodontal disease: a randomized trial. J Bodyw Mov Ther. 2025;45:536–544. doi: 10.1016/j.jbmt.2025.09.017. 41316618

[58] Ismail AMA, Tolba AMN. Effectiveness of lifestyle-modification approach (a randomized-controlled program of diet restriction and treadmill walking exercise) on elderly’s metabolic syndrome-associated subjective tinnitus. Eur Arch Otorhinolaryngol. 2025;282(8):4307–4315. doi: 10.1007/s00405-025-09494-7. 40500514 PMC12399441

[59] van der Velde JH, Savelberg HH, Schaper NC, et al. Moderate activity and fitness, not sedentary time, are independently associated with cardio-metabolic risk in U.S. adults aged 18-49. Int J Environ Res Public Health. 2015;12(3):2330–2343. doi: 10.3390/ijerph120302330. 25711356 PMC4377904

[60] Ekelund U, Steene-Johannessen J, Brown WJ, et al. Does physical activity attenuate, or even eliminate, the detrimental association of sitting time with mortality? A harmonised meta-analysis of data from more than 1 million men and women. Lancet. 2016;388(10051):1302–1310. doi: 10.1016/S0140-6736(16)30370-1. 27475271

[61] Yao K, Chen Z, Zhou W, et al. Association between hemoglobin and non-alcoholic fatty liver disease (NAFLD) in United States adults: results from NHANES 2017-2020. Prev Med Rep. 2024;44:102798. doi: 10.1016/j.pmedr.2024.102798. 38983448 PMC11231751

[62] Yu Y, Jiang L, Wang H, et al. Hepatic transferrin plays a role in systemic iron homeostasis and liver ferroptosis. Blood. 2020;136(6):726–739. doi: 10.1182/blood.2019002907. 32374849 PMC7414596

[63] Cepeda-Lopez AC, Zimmermann MB, Wussler S, et al. Greater blood volume and Hb mass in obese women quantified by the carbon monoxide-rebreathing method affects interpretation of iron biomarkers and iron requirements. Int J Obes (Lond). 2019;43(5):999–1008. doi: 10.1038/s41366-018-0127-9. 29907846 PMC6760578

