# Supplementary material for: Changes in hemoglobin levels and cardiometabolic health in adults with metabolic syndrome – a secondary outcome analysis of a six-month randomized controlled trial
Source: Ann Med. 2026 Feb 28;58(1):2635205. doi: 10.1080/07853890.2026.2635205 (PMC12951684; doi:10.1080/07853890.2026.2635205)
Supplement: Supplemental file.docx [file IANN_A_2635205_SM1935.docx]

**Supplemental materials**

**Changes in hemoglobin levels and cardiometabolic health in adults with metabolic syndrome - a secondary outcome analysis of a six-month randomized controlled trial**

Jasmina Gassner^1^, Jooa Norha^1^, Tanja Sjöros^1^, Taru Garthwaite^1^, Tiia Koivula^1^, Saara Laine^1^, Mikko Koivumäki^1^, Henri Vähä-Ypyä^2^, Petri Kallio^3,4^, Maria Saarenhovi^3^, Eliisa Löyttyniemi^5^, Harri Sievänen^2^, Noora Houttu^6^, Kirsi Laitinen^6^, Kari K. Kalliokoski^7,1^, Tommi Vasankari^2,8^, Peppi Koivunen^9^, Juhani Knuuti^1^, Ilkka Heinonen^1^

^1^Turku PET Centre, University of Turku, Åbo Akademi University, and Turku University Hospital, Turku, Finland
^2^The UKK Institute for Health Promotion Research, Tampere, Finland
^3^Department of Clinical Physiology and Nuclear Medicine, University of Turku and Turku University Hospital, Turku, Finland
^4^Paavo Nurmi Centre and Unit for Health and Physical Activity, University of Turku, Turku, Finland
^5^Department of Biostatistics, University of Turku and Turku University Hospital, Turku, Finland
^6^Institute of Biomedicine and Food and Nutrition Research Center, University of Turku, Turku, Finland
^7^Department of Biomedical Engineering, Huazhong University of Science and Technology, Wuhan, Hubei, China
^8^Faculty of Medicine and Health Technology, Tampere University, Tampere, Finland
^9^Biocenter Oulu and Faculty of Biochemistry and Molecular Medicine, Oulu Center for Cell-Matrix Research, University of Oulu, Oulu, Finland

**Contents of the supplement**

[**Supplemental Figure 1.** CONSORT Flow diagram.](#_Toc210999538)

[**Supplemental Figure 2.** Monthly distribution of participants’ enrollment dates based on the date of their first blood draw (n = 64).](#_Toc210999539)

[**Supplemental Table 1.** Baseline characteristics (n = 64).](#_Toc211007549)

[**Supplemental** **Table 2**. Correlation coefficients of baseline correlations (n = 64).](#_Toc211007550)

[**Supplemental Table 3.** Intervention effects on Hb, RBC count, HCT, MCV and MCH in the intervention and control group.](#_Toc211007551)

[**Supplemental Table 4.** Correlation coefficients between changes after 3 months (n = 61).](#_Toc211007552)

[**Supplemental Table 5.** Heatmap of correlation coefficients between changes in RBC parameters and changes in PA measures after 6-month intervention (n = 53).](#_Toc211007553)

[**Supplemental Table 6.** Pearson’s correlation coefficients between changes of RBC parameters and changes of dietary intake after 6 months (n = 57).](#_Toc211007554)

[**Supplemental Table 7.** Correlation coefficients between changes of RBC parameters and changes of metabolic parameters after 6 months (n = 58).](#_Toc211007555)

[**Supplemental Table 8**. Spearman’s rank correlation coefficients between changes of RBC parameters and changes of liver parameters after 6 months (n = 40).](#_Toc211007556)

[**Supplemental Table 9.** Pearson’s correlation coefficients between changes of RBC parameters and changes of echocardiographic parameters after 6 months (n = 55).](#_Toc211007557)

[**Supplemental Table 10.** Correlation coefficients between changes after 6 months after BMI adjustment.](#_Toc211007558)


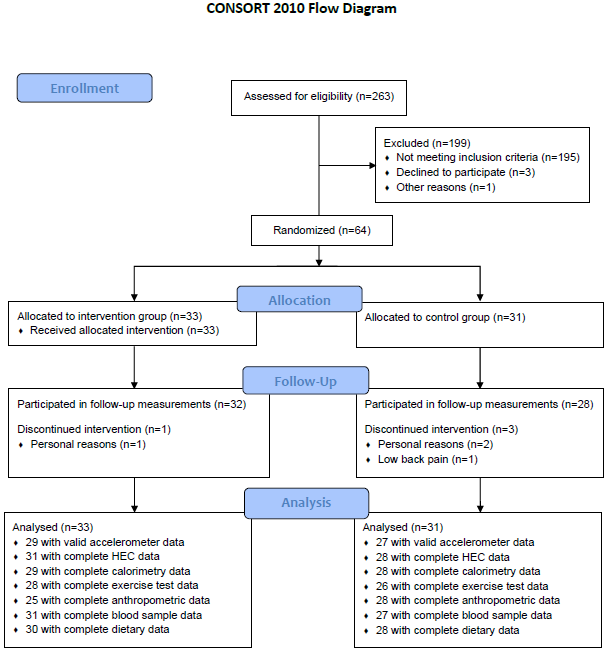


**Supplemental Figure 1.** CONSORT Flow diagram.

**Supplemental Figure 2.** Monthly distribution of participants’ enrollment dates based on the date of their first blood draw (n = 64).

**Supplemental Table 1.** Baseline characteristics (n = 64).

|  |  | Intervention | Control |
| --- | --- | --- | --- |
| Anthropometrics | |  | |
| Weight, kg | | **92.4 (16.6)** | **94.1 (15.8)** |
| BMI, kg/m2 | | 31.5 (4.0) | 31.7 (4.6) |
| Waist circumference, cm | | **111.1 (11.6)** | **110.7 (11.1)** |
| Body fat-% | | 43.1 (8.0) | 43.1 (8.0) |
| Fat mass, kg | | **39.8 (10.4)** | **40.9 (11.1)** |
| Fat-free mass, kg | | 52.6 (11.9) | 53.2 (9.8) |
| Physical activity | |  |  |
| Accelerometer wear time, h/day | | 14.5 (1.0) | 14.6 (1.0) |
| Sedentary time, %/wear time | | 69.4 (5.6) | 68.9 (6.5) |
| Standing, %/wear time | | 12.4 (3.9) | 12.1 (3.9) |
| LPA, %/wear time | | 11.6 (2.6) | 12.3 (3.0) |
| MVPA, %/wear time | | 6.64 (2.1) | 6.63 (2.3) |
| Steps/day | | 5204 (1910) | 5091 (1760) |
| Watt_max_, W/kg ^¶^ | | 1.43 (0.4) | 1.42 (0.3) |
| Watt_max_, W/kg_FFM_ ^¶^ | | 2.51 (0.5) | 2.49 (0.5) |
| VO_2_max, mL/kg/min ^\|\|^ | | 22.7 (5.0) | 22.8 (4.3) |
| VO_2_max, mL/kg_FFM_/min ^\|\|^ | | 40.0 (5.9) | 39.9 (6.4) |
| Dietary intake | |  |  |
| Energy intake, kcal/day | | 1737 (383) | 1861 (412) |
| Carbohydrates, g/day | | 165.0 (46.6) | 180.9 (50.6) |
| Fat, g/day | | 75.3 (22.8) | 83.5 (22.0) |
| Saturated fatty acids, g/day | | 26.7 (8.8) | 30.4 (8.7) |
| Monounsaturated fatty acids, g/day | | 26.5 (9.0) | 28.4 (9.6) |
| Polyunsaturated fatty acids, g/day | | 12.1 (5.0) | 13.0 (5.1) |
| Protein, g/day | | 77.4 (20.7) | 79.5 (20.0) |
| Carbohydrates, % of energy intake/day | | 38.8 (8.7) | 39.5 (6.5) |
| Fat, % of energy intake/day | | 38.2 (7.6) | 39.5 (5.1) |
| Protein, % of energy intake/day | | 18.1 (2.8) | 17.5 (2.9) |
| Metabolic parameters | |  |  |
| Fasting insulin, mU/L ^‡‡^ |  | 9 (7, 13) | 11 (7, 17) |
| Fasting glucose, mmol/L |  | 5.91 (0.5) | 5.80 (0.4) |
| HbA1c, mmol/mol | | 37.0 (2.8) | 36.3 (2.7) |
| HOMA-IR ^‡‡^ | | 2.4 (1.8, 3.8) | 2.8 (1.7, 4.9) |
| M-value, μmol·kg^−1^·min^−1^ | | 15.3 (10.7, 21.0) | 13.5 (9.8, 20.8) |
| Triglycerides, mmol/L |  | 1.54 (0.71) | 1.13 (0.47) |
| Cholesterol, mmol/L |  | 4.78 (1.01) | 4.60 (0.70) |
| LDL, mmol/L |  | 3.12 (0.85) | 3.02 (0.69) |
| HDL, mmol/L |  | 1.32 (0.32) | 1.39 (0.38) |
| Energy expenditure at rest, kcal/d ^††^ |  | 1585 (1443, 1848) | 1680 (1495, 1894) |
| O_2_ consumption at rest, ml/min ^††^ |  | 239.5 (47.5) | 240.7 (39.8) |
| ΔRespiratory exchange ratio (HEC - fasting) ^††^ | | -0.01 (-0.04, 0.02) | -0.00 (-0.04, 0.05) |
| ΔRespiratory exchange ratio (high - low intensity exercise) ^\|\|^ | | 0.40 (0.07) | 0.37 (0.07) |
| Liver parameters | |  |  |
| Liver glucose uptake, µmol/100 ml/min ^‡^ | | 2.51 (1.82, 3.31) | 2.55 (1.74, 3.11) |
| Endogenous glucose production, µmol/kg/min ^‡^ | | -1.05 (5.94) | -1.23 (10.24) |
| Liver fat content, % ^†^ | | 1.68 (1.26, 3.20) | 3.89 (0.44, 8.18) |
| ALT, U/L ^§^ | | 27 (21, 35) | 29 (20, 40) |
| AST, U/L ^§^ | | 24 (20, 31) | 22 (21, 29) |
| GGT, U/L ^§^ | | 27 (15, 39) | 22 (16, 35) |
| Echocardiographic parameters ^‡‡^ | |  |  |
| Septum thickness, mm | | 8.42 (1.3) | 8.64 (1.3) |
| LV posterior wall thickness, mm | | 8.57 (1.4) | 8.60 (1.1) |
| Relative wall thickness | | 0.32 (0.05) | 0.33 (0.05) |
| LV end-diastolic diameter, mm | | 52.9 (5.5) | 51.8 (4.1) |
| LV mass, g | | 194.2 (66.9) | 186.6 (43.0) |
| LV mass index, g/m^2^ | | 80.9 (19.3) | 78.0 (13.3) |
| Aortic root diameter, mm | | 33.1 (3.3) | 34.0 (3.9) |
| LA diameter, mm | | 39.6 (4.8) | 40.2 (4.7) |
| LA end-systolic volume index, ml/m^2 §§^ | | 27.4 (8.8) | 26.2 (6.5) |
| E peak velocity, cm/s | | 0.67 (0.14) | 0.70 (0.18) |
| A peak velocity, cm/s | | 0.77 (0.17) | 0.72 (0.15) |
| E/A ratio | | 0.91 (0.26) | 1.01 (0.29) |
| E/E' ratio | | 7.61 (1.92) | 7.40 (1.68) |
| LV end-diastolic volume, ml | | 88.9 (25.8) | 91.6 (18.3) |
| LV end-systolic volume, ml | | 35.3 (16.5) | 34.8 (10.1) |
| LV ejection fraction, % | | 63.0 (4.3) | 62.8 (4.8) |
| LV stroke volume, ml | | 53.6 (14.6) | 56.8 (11.8) |
| Cardiac output, ml/min | | 3757 (943) | 4071 (870) |
| Global longitudinal strain, % ^¶^ | | -18.0 (2.9) | -18.9 (2.2) |
| Global longitudinal strain - exercise 25W ^n=45^ | | -18.0 (3.0) | -18.9 (2.8) |
| Global longitudinal strain - exercise 50W ^n=42^ | | -17.9 (3.6) | -19.0 (2.8) |
| Global longitudinal strain - exercise 75W ^n=38^ | | -19.2 (3.0) | -18.7 (3.2) |
| Global longitudinal strain - exercise 100W ^n=32^ | | -17.9 (3.9) | -18.9 (3.6) |
| Global longitudinal strain - exercise 125W ^n=25^ | | -15.6 (3.5) | -18.2 (4.5) |

BMI = body mass index; LPA = light-intensity physical activity; MVPA = moderate-to-vigorous physical activity; VO_2_max = maximal oxygen consumption; FFM = fat free mass; HOMA-IR = Homeostatic Model Assessment for Insulin Resistance; M-value = whole-body glucose uptake in hyperinsulinemic euglycemic clamp (HEC); LDL = low density lipoprotein; HDL = high density lipoprotein; ALT = alanine aminotransferase; AST = aspartate aminotransferase; GGT = γ-glutamyltransferase; LV = Left ventricle; LA = left atrium; E = peak early diastolic filling velocity; A = peak late (atrial) diastolic filling velocity; E’ = lateral early diastolic mitral annulus velocity.

Values are presented as mean (SD) or Median (Q1, Q3) for non-normally distributed data.

Data available for: † = 40; ‡ = 43; § = 44; || = 58; ¶ = 59; §§ = 60; †† = 62; ‡‡ = 63 participants.

**Supplemental** **Table 2**. Correlation coefficients of baseline correlations (n = 64).

|  | **Hb, g/L** | **HCT, %** | **RBC count, 10^12^/L** | **MCV, fl** | **MCH, pg** |
| --- | --- | --- | --- | --- | --- |
| **Anthropometrics** |  |  |  |  |  |
| Weight, kg | **0.27*** | 0.23 | **0.32*** | **-0.25*** | -0.12 |
| BMI, kg/m^2^ | 0.04 | 0.04 | 0.05 | -0.03 | -0.01 |
| Waist circumference, cm | **0.27*** | **0.27*** | **0.27*** | -0.08 | -0.06 |
| Body fat-% | **-0.31*** | **-0.28*** | **-0.36**** | **0.27*** | 0.14 |
| Fat mass, kg | -0.04 | -0.05 | -0.05 | 0.02 | 0.03 |
| Fat-free mass, kg | **0.44**** | **0.39**** | **0.52**** | **-0.40**** | -0.20 |
| **Physical activity** |  |  |  |  |  |
| Sedentary time, %/wear time | 0.23 | 0.23 | **0.31*** | **-0.28*** | -0.19 |
| Standing, %/wear time | **-0.35**** | **-0.35**** | **-0.40**** | 0.22 | 0.17 |
| LPA, %/wear time | -0.04 | -0.02 | -0.17 | **0.37**** | **0.26*** |
| MVPA, %/wear time | 0.04 | 0.02 | 0.09 | -0.09 | -0.10 |
| Steps/day | -0.07 | -0.07 | -0.01 | -0.06 | -0.11 |
| Watt_max_, W/kg ^¶^ | 0.08 | 0.01 | 0.00 | -0.02 | 0.10 |
| Watt_max_, W/kg_FFM_ ^¶^ | -0.12 | -0.17 | -0.22 | 0.14 | 0.19 |
| VO_2_max, mL/kg/min  ^\|\|^ | 0.12 | 0.04 | 0.03 | -0.02 | 0.14 |
| VO_2_max, mL/kg_FFM_/min  ^\|\|^ | -0.07 | -0.13 | -0.21 | 0.18 | 0.24 |
| **Dietary intake** |  |  |  |  |  |
| Energy intake, kcal/day | **0.28*** | 0.21 | 0.18 | 0.02 | 0.15 |
| Protein, g/day | 0.20 | 0.16 | 0.18 | -0.07 | 0.04 |
| Carbohydrates, g/day | **0.25*** | 0.18 | 0.15 | 0.02 | 0.17 |
| Fat, g/day | 0.15 | 0.11 | 0.10 | 0.02 | 0.09 |
| Saturated fatty acids, g/day | 0.09 | 0.02 | 0.01 | 0.04 | 0.13 |
| Monounsaturated fatty acids, g/day | 0.18 | 0.15 | 0.13 | 0.02 | 0.06 |
| Polyunsaturated fatty acids, g/day | 0.21 | 0.20 | 0.16 | 0.01 | 0.08 |
| Protein, % of energy intake/d | -0.05 | -0.02 | 0.04 | -0.14 | -0.17 |
| Carbohydrates, % of energy intake/d | 0.03 | -0.01 | -0.05 | 0.07 | 0.13 |
| Fat, % of energy intake/d | -0.05 | -0.03 | -0.01 | -0.02 | -0.08 |
| Saturated fatty acids, % of energy intake/d | -0.12 | -0.14 | -0.13 | 0.03 | 0.03 |
| Monounsaturated fatty acids, % of energy intake /d | 0.03 | 0.07 | 0.08 | -0.03 | -0.10 |
| Polyunsaturated fatty acids, % of energy intake /d | 0.10 | 0.13 | 0.11 | -0.02 | -0.02 |
| **Metabolic parameters** |  |  |  |  |  |
| VO_2_ at rest, ml/min ^§§^ | **0.41**** | **0.39**** | **0.50***** | **-0.33**** | -0.21 |
| Energy expenditure at rest, kcal/d ^§§^ ^rs^ | **0.49***** | **0.47***** | **0.53***** | -0.24 | -0.02 |
| ΔRER (HEC-fasting) ^§§ rs^ | -0.14 | -0.14 | -0.15 | 0.08 | -0.06 |
| ΔRER (high-low intensity exercise)  ^\|\|^ | **-0.34**** | **-0.26*** | **-0.32*** | 0.21 | 0.00 |
| M-value, μmol·kg^−1^·min^−1 rs^ | **-0.36**** | **-0.35**** | **-0.44***** | 0.24 | 0.13 |
| Fasting glucose, mmol/L | 0.18 | 0.19 | 0.21 | -0.06 | -0.09 |
| Fasting insulin, mU/L ^\|\|\|\| rs^ | 0.25 | 0.24 | **0.33**** | -0.19 | -0.05 |
| HOMA-IR ^\|\|\|\| rs^ | **0.27*** | **0.27*** | **0.34**** | -0.18 | -0.03 |
| HbA1c, mmol/mol | 0.04 | 0.11 | 0.07 | 0.05 | -0.07 |
| Triglycerides, mmol/L | 0.15 | 0.11 | 0.11 | -0.04 | 0.03 |
| Cholesterol, mmol/L | -0.11 | -0.06 | -0.17 | **0.25*** | 0.14 |
| LDL, mmol/L | -0.07 | -0.05 | -0.09 | 0.12 | 0.06 |
| HDL, mmol/L | -0.22 | -0.14 | **-0.28*** | **0.35**** | 0.16 |
| **Liver parameters** |  |  |  |  |  |
| Liver glucose uptake, µmol/100 ml/min ^¶ rs^ | -0.19 | -0.15 | **-0.34*** | **0.39*** | 0.19 |
| Endogenous glucose production, µmol/kg/min ^¶^ | -0.02 | 0.01 | 0.13 | -0.25 | **-0.32*** |
| Liver fat content, % ^† rs^ | **0.40*** | **0.37*** | 0.26 | 0.24 | 0.26 |
| ALT, U/L ^§ rs^ | 0.29 | 0.29 | 0.29 | 0.00 | 0.08 |
| AST, U/L  ^§ rs^ | 0.08 | 0.13 | 0.13 | 0.08 | 0.05 |
| GGT, U/L  ^§ rs^ | 0.28 | **0.33*** | **0.32*** | 0.02 | 0.01 |
| **Echocardiographic parameters** ^\|\|\|\|^ |  |  |  |  |  |
| Septum thickness, mm | **0.34**** | **0.30*** | **0.39**** | **-0.28*** | -0.15 |
| LV posterior wall thickness, mm | **0.42**** | **0.36**** | **0.45***** | **-0.32*** | -0.12 |
| Relative wall thickness | **0.34**** | **0.29*** | **0.33**** | -0.19 | -0.04 |
| LV end-diastolic diameter, mm | 0.12 | 0.08 | 0.16 | -0.18 | -0.09 |
| LV mass, g | **0.32*** | **0.29*** | **0.38**** | **-0.31*** | -0.17 |
| LV mass index, g/m^2^ | **0.29*** | **0.26*** | **0.32**** | -0.23 | -0.11 |
| Aortic root diameter, mm | **0.34**** | **0.35**** | **0.44***** | **-0.31*** | -0.22 |
| LA diameter, mm | 0.12 | 0.10 | 0.09 | 0.04 | 0.03 |
| LA end-systolic volume index, ml/m^2 ††^ | -0.07 | -0.04 | -0.01 | -0.06 | -0.08 |
| E peak velocity, cm/s | -0.09 | -0.07 | -0.01 | -0.12 | -0.13 |
| A peak velocity, cm/s | -0.04 | -0.03 | -0.04 | 0.09 | 0.00 |
| E/A ratio | -0.06 | -0.06 | 0.03 | -0.21 | -0.15 |
| E/E' ratio | 0.01 | 0.08 | 0.13 | -0.15 | -0.22 |
| LV end-diastolic volume, ml | 0.11 | 0.11 | 0.20 | -0.265* | -0.18 |
| LV end-systolic volume, ml | -0.03 | -0.02 | 0.13 | **-0.34**** | **-0.33**** |
| LV ejection fraction, % | 0.01 | 0.01 | 0.01 | -0.01 | 0.03 |
| LV stroke volume, ml | 0.22 | 0.20 | 0.20 | -0.10 | 0.03 |
| Cardiac output, ml/min ^‡‡^ | 0.15 | 0.13 | 0.17 | -0.15 | -0.04 |
| Global longitudinal strain, % ^¶^ | **0.27*** | 0.25 | 0.23 | -0.05 | 0.00 |
| GLS exercise 25W ^n=44^ | -0.14 | -0.11 | -0.03 | -0.11 | -0.16 |
| GLS exercise 50W ^n=42^ | **-0.43**** | **-0.37*** | **-0.33*** | 0.04 | -0.10 |
| GLS exercise 75W ^n=38^ | **-0.40*** | -0.32 | -0.30 | 0.08 | -0.08 |
| GLS exercise 100W^n=32^ | -0.30 | -0.27 | -0.33 | 0.28 | 0.11 |
| GLS exercise 125W ^n=25^ | -0.36 | -0.33 | -0.23 | -0.17 | -0.20 |

Hb = Hemoglobin; HCT = Hematocrit; RBC = red blood cell; MVC = mean corpuscular volume; MCH = mean corpuscular hemoglobin; BMI = body mass index; LPA = light-intensity physical activity; MVPA = moderate-to-vigorous physical activity; VO_2_max = maximal oxygen consumption; FFM = fat free mass; VO_2_ = oxygen consumption; RER = respiratory exchange ratio; HOMA-IR = Homeostatic Model Assessment for Insulin Resistance; M-value = whole-body glucose uptake in hyperinsulinemic euglycemic clamp (HEC); LDL = low density lipoprotein; HDL = high density lipoprotein; ALT = alanine aminotransferase; AST = aspartate aminotransferase; GGT = γ-glutamyltransferase; LV = Left ventricle; LA = left atrium; E = peak early diastolic filling velocity; A = peak late (atrial) diastolic filling velocity; E’ = lateral early diastolic mitral annulus velocity; GLS = global longitudinal strain.

Pearson’s correlation was performed unless data deviated from normality, in which case Spearman’s rank correlation (marked with = rs) was used.
Bold values indicate statistical significance: * = p < 0.05, ** = p < 0.01, ***=p < 0.001.

†=n=40; ‡=n=43; §=n=44; ||=n=58; ¶=n=59; ††=n= 60; ‡‡=n=61; §§=n=62; ||||=n=63.

**Supplemental Table 3.** Intervention effects on Hb, RBC count, HCT, MCV and MCH in the intervention and control group.

|  | Intervention group | | | Control group | | | p-values | | |
| --- | --- | --- | --- | --- | --- | --- | --- | --- | --- |
|  | Baseline | 3 months | 6 months | Baseline | 3 months | 6 months | Group | Time | group*time |
| Hb, g/L | 141.8 (138.5, 145.2) | 149.0 (145.6, 152.3) | 140.0 (136.6, 143.4) | 140.0 (136.3, 143.1) | 147.0 (143.6, 150.5) | 139.1 (135.6, 142.7) | 0.4601 | **<0.0001** | 0.7527 |
| RBC count, 10^12^/L | 4.696 (4.579, 4.813) | 4.940 (4.824, 5.057) | 4.662 (4.544, 4.780) | 4.591 (4.471, 4.710) | 4.839 (4.719, 4.960) | 4.563 (4.440, 4.685) | 0.2291 | **<0.0001** | 0.8041 |
| HTC, % | 42.0 (41.1, 42.9) | 44.3 (43.4, 45.3) | 41.8 (40.8, 42.7) | 41.1 (40.2, 42.1) | 43.6 (42.6, 44.5) | 41.2 (40.3, 42.2) | 0.1932 | **<0.0001** | 0.9938 |
| MCV, fl | 89.6 (88.2, 90.9) | 89.8 (88.5, 91.1) | 89.7 (88.4, 91.0) | 89.6 (88.3, 91.0) | 90.1 (88.7, 91.4) | 90.7 (89.4, 92.1) | 0.6164 | **0.0213** | 0.1106 |
| MCH, pg | 30.3 (29.8, 30.8) | 30.3 (29.7, 30.9) | 30.3 (29.7, 30.8) | 30.58 (30.0, 31.0) | 30.6 (30.0, 31.2) | 30.6 (30.0, 31.2) | 0.4712 | 0.9926 | 0.7842 |

Hb = Hemoglobin; RBC = red blood cell; HCT = Hematocrit; MVC = mean corpuscular volume; MCH = mean corpuscular hemoglobin.

Values are presented as model-based means and 95% CIs.

**Supplemental Table 4.** Correlation coefficients between changes after 3 months (n = 61).

|  | Δ**Hb, g/L** | Δ**HCT, %** | Δ**RBC count, 10^12^/l ^rs^** | Δ**MCV, fl** | Δ**MCH, pg** |
| --- | --- | --- | --- | --- | --- |
| **Anthropometrics** |  |  |  |  |  |
| ΔWeight, kg | -0.02 | 0.01 | -0.04 | -0.09 | 0.10 |
| ΔBMI, kg/m^2^ | -0.01 | 0.01 | -0.04 | -0.08 | 0.11 |
| ΔWaist circumference, cm ^†^ | -0.14 | -0.04 | -0.02 | 0.04 | -0.03 |
| ΔBody fat-% | 0.08 | 0.04 | 0.17 | **-0.29*** | **-0.27*** |
| ΔFat mass, kg ^rs^ | 0.06 | 0.03 | 0.10 | **-0.30*** | -0.19 |
| ΔFat-free mass, kg | -0.13 | -0.10 | -0.23 | **0.26*** | **0.39**** |
| **Physical activity ‡** |  |  |  |  |  |
| ΔSedentary time, %/wear time | -0.04 | -0.05 | -0.11 | -0.07 | -0.04 |
| ΔStanding, %/wear time | 0.09 | 0.10 | 0.13 | 0.12 | 0.07 |
| ΔLPA, %/wear time | -0.10 | -0.09 | 0.09 | -0.08 | -0.13 |
| ΔMVPA, %/wear time | 0.06 | 0.06 | 0.08 | 0.06 | 0.11 |
| ΔSteps/day ^rs^ | 0.17 | 0.12 | 0.12 | -0.01 | 0.13 |
| **Metabolic parameters** |  |  |  |  |  |
| ΔFasting glucose, mmol/L | **0.28*** | 0.23 | 0.21 | 0.04 | 0.07 |
| ΔFasting insulin, mU/L | 0.16 | 0.21 | 0.09 | 0.07 | 0.02 |
| ΔHOMA-IR | 0.20 | 0.24 | 0.14 | 0.10 | 0.05 |
| ΔHbA1c, mmol/mol | 0.01 | 0.07 | 0.06 | -0.18 | 0.01 |
| ΔTriglycerides, mmol/L | -0.08 | 0.00 | 0.02 | 0.03 | -0.02 |
| ΔCholesterol, mmol/L | 0.03 | 0.06 | 0.01 | 0.05 | 0.11 |
| ΔLDL, mmol/L | 0.13 | 0.06 | 0.05 | 0.00 | 0.18 |
| ΔHDL, mmol/L | 0.20 | 0.16 | 0.17 | -0.01 | 0.02 |

Hb = Hemoglobin; HCT = Hematocrit; RBC = red blood cell; MVC = mean corpuscular volume; MCH = mean corpuscular hemoglobin; BMI = body mass index; LPA = light-intensity physical activity; MVPA = moderate-to-vigorous physical activity; HOMA-IR = Homeostatic Model Assessment for Insulin Resistance; LDL = low density lipoprotein; HDL = high density lipoprotein.
Pearson’s correlation was performed unless data deviated from normality, in which case Spearman’s rank correlation (marked with = rs) was used.

Bold values indicate statistical significance: * = p < 0.05, ** = p < 0.01, *** = p < 0.001.
† = n = 59, ‡ = n = 48.

**Supplemental Table 5.** Heatmap of correlation coefficients between changes in RBC parameters and changes in PA measures after 6-month intervention (n = 53).

| **Physical activity** | ΔHb, g/L | ΔHct, % | ΔRBC count,  10^12^/L | ΔMCV, fl | ΔMCH, pg |
| --- | --- | --- | --- | --- | --- |
| ΔSedentary time, %/wear time † | 0.14 | 0.15 | 0.12 | 0.07 | -0.08 |
| ΔStanding, %/wear time † | -0.14 | -0.14 | -0.11 | -0.09 | 0.01 |
| ΔLPA, %/wear time † | -0.18 | -0.22 | -0.16 | -0.1 | 0.01 |
| ΔMVPA, %/wear time † | 0.05 | 0.03 | -0.00 | 0.06 | 0.18 |
| ΔSteps/day † rs | 0.05 | -0.05 | -0.09 | 0.16 | **0.42**** |
| ΔWattmax, W/kg rs | **-0.29*** | -0.19 | -0.26 | 0.08 | 0.02 |
| ΔWattmax, W/kgFFM rs | **-0.38**** | -0.26 | **-0.31*** | 0.02 | -0.06 |
| ΔVO2max, mL/kg/min ‡ | -0.04 | 0 | -0.09 | 0.12 | 0.07 |
| ΔVO2max, mL/kgFFM/min ‡ | -0.06 | -0.04 | -0.09 | 0.01 | 0.05 |

Hb = Hemoglobin; HCT = Hematocrit; RBC = red blood cell; MVC = mean corpuscular volume; MCH = mean corpuscular hemoglobin; LPA = light-intensity physical activity; MVPA = moderate-to-vigorous physical activity
Pearson’s correlation was performed unless data deviated from normality, in which case Spearman’s rank correlation (marked with = rs) was used.
Red indicates positive and blue negative correlation. Bold values indicate statistical significance: * = p < 0.05, ** = p < 0.01. † = n = 51, ‡ = n = 52.

**Supplemental Table 6.** Pearson’s correlation coefficients between changes of RBC parameters and changes of dietary intake after 6 months (n = 57).

|  | Δ**Hb, g/L** | Δ**HCT, %** | Δ**RBC count, 10^12^/l** | Δ**MCV, fl** | Δ**MCH, pg** |
| --- | --- | --- | --- | --- | --- |
| **Dietary intake** |  |  |  |  |  |
| ΔEnergy intake, kcal/day | 0.07 | 0.10 | 0.12 | -0.03 | -0.10 |
| ΔProtein, g/day | 0.07 | 0.16 | 0.14 | 0.06 | -0.15 |
| ΔCarbohydrates, g/day | 0.01 | 0.03 | -0.01 | 0.05 | 0.10 |
| ΔFat, g/day | 0.10 | 0.12 | 0.18 | -0.11 | -0.24 |
| ΔSaturated fatty acids, g/day | 0.12 | 0.14 | 0.17 | -0.04 | -0.12 |
| ΔMonounsaturated fatty acids, g/day | 0.19 | 0.20 | **0.32*** | -0.24 | **-0.30*** |
| ΔPolyunsaturated fatty acids, g/day | 0.24 | **0.26*** | **0.28*** | -0.07 | -0.11 |
| ΔProtein, % of energy intake/d | -0.02 | 0.03 | -0.01 | 0.07 | -0.04 |
| ΔCarbohydrates, % of energy intake/d | -0.13 | -0.11 | -0.16 | 0.06 | 0.19 |
| ΔFat, % of energy intake/d | 0.12 | 0.13 | 0.16 | -0.06 | -0.18 |
| ΔSaturated fatty acids, % of energy intake/d | 0.10 | 0.10 | 0.09 | 0.04 | 0.00 |
| ΔMonounsaturated fatty acids, % of energy intake /d | 0.25 | 0.26 | **0.38**** | -0.26 | **-0.29*** |
| ΔPolyunsaturated fatty acids, % of energy intake /d | **0.26*** | 0.26 | 0.25 | -0.04 | -0.03 |

Hb = Hemoglobin; HCT = Hematocrit; RBC = red blood count; MVC = mean corpuscular volume; MCH = mean corpuscular hemoglobin.

Bold values indicate statistical significance: * = p < 0.05, ** = p < 0.01.

**Supplemental Table 7.** Correlation coefficients between changes of RBC parameters and changes of metabolic parameters after 6 months (n = 58).

|  | Δ**Hb, g/L** | Δ**HCT, %** | Δ**RBC count, 10^12^/L** | Δ**MCV, fl** | Δ**MCH, pg** |
| --- | --- | --- | --- | --- | --- |
| **Metabolic parameters** |  |  |  |  |  |
| ΔVO_2_ at rest, ml/min ^‡^ | -0.01 | 0.12 | 0.05 | -0.01 | -0.12 |
| ΔEnergy expenditure at rest, kcal/d ^‡^ | -0.03 | 0.09 | 0.03 | 0.01 | -0.13 |
| Δ (ΔRER (HEC-fasting)) ^‡ rs^ | -0.02 | 0.02 | 0.01 | -0.09 | -0.04 |
| Δ (ΔRER (high-low exercise)) ^†^ ^rs^ | -0.04 | -0.01 | -0.13 | 0.11 | -0.01 |
| ΔM-value, μmol·kg^−1^·min^−1 rs^ | **-0.26*** | -0.18 | -0.21 | -0.04 | 0.02 |
| ΔFasting glucose, mmol/L | **0.29*** | **0.28*** | **0.29*** | 0.02 | -0.06 |
| ΔFasting insulin, mU/L | 0.20 | 0.22 | 0.09 | 0.21 | 0.15 |
| ΔHOMA-IR ^rs^ | 0.26 | **0.28*** | 0.20 | 0.16 | -0.05 |
| ΔHbA1c, mmol/mol ^§^ | -0.06 | -0.08 | 0.10 | **-0.45***** | -0.26 |
| ΔTriglycerides, mmol/L | 0.09 | 0.11 | 0.09 | -0.01 | -0.01 |
| ΔCholesterol, mmol/L | 0.14 | 0.13 | -0.01 | 0.23 | 0.19 |
| ΔLDL, mmol/L | -0.10 | -0.11 | -0.15 | 0.07 | 0.09 |
| ΔHDL, mmol/L ^rs^ | **0.28*** | **0.28*** | **0.28*** | 0.06 | -0.01 |

RBC = red blood count; Hb = Hemoglobin; HCT = Hematocrit; MVC = mean corpuscular volume; MCH = mean corpuscular hemoglobin; VO_2_ = oxygen consumption; RER = respiratory exchange ratio. HOMA-IR = Homeostatic Model Assessment for Insulin Resistance; M-value = whole-body glucose uptake in hyperinsulinemic euglycemic clamp (HEC); LDL = low density lipoprotein; HDL = high density lipoprotein.
Pearson’s correlation was performed unless data deviated from normality, in which case Spearman’s rank correlation (marked with = rs) was used.
Bold values indicate statistical significance: * = p < 0.05, ** = p < 0.01, *** = p < 0.001.
†=n=52; ‡=n=56; §=n=57.

**Supplemental Table 8**. Spearman’s rank correlation coefficients between changes of RBC parameters and changes of liver parameters after 6 months (n = 40).

|  | Δ**Hb, g/L** | Δ**HCT, %** | Δ**RBC count, 10^12^/L** | Δ**MCV, fl** | Δ**MCH, pg** |
| --- | --- | --- | --- | --- | --- |
| **Liver parameters** |  |  |  |  |  |
| ΔLiver glucose uptak, µmol/100 ml/min ^†^ | 0.07 | 0.18 | 0.15 | -0.02 | -0.12 |
| ΔEndogenous glucose production, µmol/kg/min ^‡^ | 0.08 | 0.05 | 0.05 | -0.05 | -0.07 |
| ΔLiver fat content, % ^§^ | 0.27 | 0.24 | **0.36*** | -0.01 | -0.23 |
| ΔALT, U/L | 0.02 | 0.05 | -0.03 | -0.11 | 0.06 |
| ΔAST, U/L | 0.07 | 0.08 | 0.00 | 0.01 | 0.29 |
| ΔGGT, U/L | 0.00 | 0.02 | 0.05 | -0.09 | -0.04 |

Hb = Hemoglobin; HCT = Hematocrit; RBC = red blood cell; MVC = mean corpuscular volume; MCH = mean corpuscular hemoglobin; ALT = alanine aminotransferase; AST = aspartate aminotransferase; GGT = γ-glutamyltransferase.

Bold values indicate statistical significance: * = p < 0.05. †=n=38; ‡=n=39; §=n=34.

**Supplemental Table 9.** Pearson’s correlation coefficients between changes of RBC parameters and changes of echocardiographic parameters after 6 months (n = 55).

|  | **ΔHb, g/L** | **ΔHCT, %** | **ΔRBC count, 10^12^/L** | **ΔMCV, fl** | **ΔMCH, pg** |
| --- | --- | --- | --- | --- | --- |
| **Echocardiographic parameters** |  |  |  |  |  |
| ΔSeptum thickness, mm | 0.16 | 0.21 | 0.02 | 0.26 | 0.21 |
| ΔLV posterior wall thickness, mm | -0.07 | -0.11 | 0.09 | **-0.44***** | -0.22 |
| ΔRelative wall thickness | -0.18 | -0.19 | 0.03 | **-0.50***** | **-0.32*** |
| ΔLV end-diastolic diameter, mm | 0.25 | 0.21 | 0.05 | **0.36**** | **0.37**** |
| ΔLV mass, g | 0.18 | 0.19 | 0.03 | 0.24 | 0.24 |
| ΔLV mass index, g/m^2^ | 0.25 | 0.23 | 0.10 | 0.19 | 0.22 |
| ΔAortic root diameter, mm | 0.12 | 0.15 | 0.14 | 0.02 | -0.10 |
| ΔLA diameter, mm ^††^ | 0.05 | 0.09 | -0.01 | 0.18 | 0.04 |
| ΔLA end-systolic volume index, ml/m^2^ ^¶^ | 0.05 | 0.13 | 0.14 | -0.14 | -0.21 |
| ΔE peak velocity cm/s | 0.08 | 0.08 | 0.07 | 0.06 | -0.17 |
| ΔA peak velocity cm/s | 0.04 | -0.02 | -0.02 | 0.04 | -0.04 |
| ΔE/A ratio | 0.04 | 0.14 | 0.12 | -0.01 | -0.18 |
| ΔE/E' ratio ^††^ | 0.05 | 0.01 | 0.03 | -0.15 | -0.04 |
| ΔLV end-diastolic volume, ml | 0.15 | 0.09 | -0.03 | 0.16 | 0.17 |
| ΔLV end-systolic volume, ml | 0.00 | 0.00 | -0.17 | **0.33*** | 0.19 |
| ΔLV ejection fraction, % | 0.13 | 0.14 | 0.08 | 0.14 | 0.14 |
| ΔLV stroke volume, ml | 0.21 | 0.12 | 0.14 | -0.12 | 0.04 |
| ΔCardiac output, ml/min ^¶^ | 0.16 | 0.06 | 0.10 | -0.16 | 0.07 |
| ΔGlobal longitudinal strain, % | 0.09 | 0.05 | 0.11 | -0.13 | 0.09 |
| ΔGLS exercise 25W ^\|\|^ | -0.06 | 0.01 | -0.04 | 0.14 | 0.05 |
| ΔGLS exercise 50W ^§^ | -0.02 | 0.02 | 0.00 | 0.09 | -0.02 |
| ΔGLS exercise 75W ^§^ | 0.31 | 0.25 | 0.19 | 0.01 | 0.25 |
| ΔGLS exercise 100W ^⁑^ | -0.20 | -0.27 | -0.34 | 0.04 | 0.06 |
| ΔGLS exercise 125W ^†^ | 0.02 | 0.02 | 0.10 | -0.24 | -0.41 |

Hb = Hemoglobin; HCT = Hematocrit; RBC = red blood cell; MVC = mean corpuscular volume; MCH = mean corpuscular hemoglobin; LV = Left ventricle; LA = left atrium; E = peak early diastolic filling velocity; A = peak late (atrial) diastolic filling velocity; E’ = lateral early diastolic mitral annulus velocity; GLS = global longitudinal strain.

Bold values indicate statistical significance: * = p < 0.05, ** = p < 0.01, *** = p < 0.001.
†=n=14; ⁑=n=22; §=n=30; ||=n=31; ¶=n=52; ††=n=54.

**Supplemental Table 10.** Correlation coefficients between changes after 6 months after BMI adjustment.

|  | **Hb, g/L** | **HCT, %** | **RBC count, 10^12^/L** | **MCV, fl** | **MCH, pg** |
| --- | --- | --- | --- | --- | --- |
| **Anthropometrics** |  |  |  |  |  |
| ΔWeight, kg | -0.13 | -0.19 | -0.20 | -0.02 | 0.16 |
| ΔWaist circumference, cm | -0.24 | -0.22 | -0.26 | -0.01 | 0.05 |
| ΔBody fat-% | -0.03 | -0.05 | 0.03 | -0.24 | -0.01 |
| ΔFat mass, kg | -0.05 | -0.08 | 0.01 | -0.26 | -0.01 |
| ΔFat-free mass, kg | 0.07 | 0.05 | -0.01 | **0.28*** | 0.08 |
| **Physical activity** |  |  |  |  |  |
| ΔSedentary time, %/wear time | 0.07 | 0.07 | 0.05 | 0.07 | -0.04 |
| ΔStanding, %/wear time | -0.12 | -0.11 | -0.08 | -0.09 | -0.00 |
| ΔLPA, %/wear time | -0.09 | -0.12 | -0.06 | -0.10 | -0.04 |
| ΔMVPA, %/wear time | 0.10 | 0.10 | 0.06 | 0.07 | 0.16 |
| ΔSteps/day | 0.14 | 0.07 | -0.01 | 0.17 | **0.38**** |
| ΔWatt_max_, W/kg ^††^ | -0.22 | -0.10 | -0.18 | 0.08 | -0.04 |
| ΔWatt_max_, W/kg_FFM_ ^††^ | **-0.33*** | -0.18 | -0.24 | 0.01 | -0.12 |
| ΔVO_2_max, mL/kg/min ^¶^ | 0.04 | 0.10 | -0.01 | 0.13 | 0.04 |
| ΔVO_2_max, mL/kg_FFM_/min ^¶^ | 0.01 | 0.05 | -0.02 | 0.02 | 0.02 |
| **Dietary intake** |  |  |  |  |  |
| ΔEnergy intake, kcal/day | -0.02 | -0.01 | 0.03 | -0.03 | -0.06 |
| ΔProtein, g/day | -0.01 | 0.06 | 0.06 | 0.06 | -0.12 |
| ΔCarbohydrates, g/day | -0.07 | -0.07 | -0.09 | 0.05 | 0.15 |
| ΔFat, g/day | 0.04 | 0.05 | 0.12 | -0.11 | -0.21 |
| ΔSaturated fatty acids, g/day | 0.10 | 0.12 | 0.15 | -0.04 | -0.11 |
| ΔMonounsaturated fatty acids, g/day | 0.10 | 0.09 | 0.24 | **-0.27*** | **-0.28*** |
| ΔPolyunsaturated fatty acids, g/day | 0.17 | 0.17 | 0.21 | -0.08 | -0.08 |
| ΔProtein, % of energy intake/d | -0.02 | 0.03 | -0.01 | 0.07 | -0.04 |
| ΔCarbohydrates, % of energy intake/d | -0.17 | -0.16 | -0.20 | 0.06 | 0.21 |
| ΔFat, % of energy intake/d | 0.14 | 0.16 | 0.18 | -0.06 | -0.19 |
| ΔSaturated fatty acids, % of energy intake/d | 0.16 | 0.17 | 0.15 | 0.05 | -0.02 |
| ΔMonounsaturated fatty acids, % of energy intake /d | 0.21 | 0.21 | **0.34*** | **-0.27*** | **-0.27*** |
| ΔPolyunsaturated fatty acids, % of energy intake /d | 0.23 | 0.22 | 0.22 | -0.05 | -0.01 |
| **Metabolic parameters** |  |  |  |  |  |
| ΔVO_2_ at rest, ml/min ^§§^ | -0.15 | -0.03 | -0.08 | -0.01 | -0.08 |
| ΔEnergy expenditure at rest, kcal/d ^§§^ | -0.18 | -0.07 | -0.12 | 0.01 | -0.08 |
| Δ(ΔRER (HEC-fasting)) ^§§^ | 0.01 | 0.05 | 0.04 | -0.09 | -0.05 |
| Δ(ΔRER (high-low intensity exercise)) ^¶^ | 0.06 | 0.09 | -0.06 | 0.11 | -0.06 |
| ΔM-value, μmol·kg^−1^·min^−1^ | -0.15 | -0.04 | -0.09 | -0.06 | -0.07 |
| ΔFasting glucose, mmol/L | 0.24 | 0.23 | 0.25 | 0.02 | -0.03 |
| ΔFasting insulin, mU/L ^\|\|\|\|^ | 0.17 | 0.20 | 0.11 | 0.19 | -0.01 |
| ΔHOMA-IR ^\|\|\|\|^ | 0.19 | 0.21 | 0.13 | 0.17 | -0.01 |
| ΔHbA1c, mmol/mol | -0.15 | -0.20 | 0.01 | **-0.48***** | -0.23 |
| ΔTriglycerides, mmol/L | 0.06 | 0.07 | 0.05 | -0.02 | 0.01 |
| Cholesterol, mmol/L | 0.11 | 0.10 | -0.05 | 0.23 | 0.21 |
| ΔLDL, mmol/L | -0.10 | -0.12 | -0.16 | 0.07 | 0.09 |
| ΔHDL, mmol/L | **0.27*** | **0.26*** | **0.26*** | 0.06 | 0.01 |
| **Liver parameters** |  |  |  |  |  |
| ΔLiver glucose uptake, µmol/100 ml/min ^‡^ | 0.07 | 0.18 | 0.15 | -0.02 | -0.12 |
| ΔEndogenous glucose production, µmol/kg/min ^‡^ | 0.05 | 0.00 | 0.01 | -0.05 | -0.07 |
| ΔLiver fat content, % ^†^ | 0.27 | 0.20 | 0.30 | -0.01 | -0.20 |
| ΔALT, U/L ^§^ | -0.02 | 0.02 | -0.06 | -0.10 | 0.08 |
| ΔAST, U/L ^§^ | 0.09 | 0.07 | 0.02 | 0.01 | 0.30 |
| ΔGGT, U/L ^§^ | 0.01 | 0.01 | 0.05 | -0.09 | -0.04 |
| **Echocardiographic parameters** ^\|\|\|\|^ |  |  |  |  |  |
| ΔSeptum thickness, mm | 0.15 | 0.18 | 0.07 | 0.16 | 0.17 |
| ΔLV posterior wall thickness, mm | -0.06 | -0.13 | 0.08 | **-0.44***** | -0.19 |
| ΔRelative wall thickness | -0.14 | -0.18 | 0.06 | **-0.50***** | -0.26 |
| ΔLV end-diastolic diameter, mm | **0.28*** | 0.25 | 0.09 | **0.32*** | **0.36**** |
| ΔLV mass, g | 0.10 | 0.12 | 0.07 | 0.04 | 0.10 |
| ΔLV mass index, g/m^2^ | 0.17 | 0.13 | 0.09 | 0.06 | 0.14 |
| ΔAortic root diameter, mm | 0.17 | 0.20 | 0.19 | 0.10 | -0.01 |
| ΔLA diameter, mm | 0.12 | 0.10 | 0.09 | 0.04 | 0.03 |
| ΔLA end-systolic volume index, ml/m^2 ‡‡^ | 0.04 | 0.15 | 0.14 | -0.06 | -0.14 |
| ΔE peak velocity, cm/s | -0.03 | -0.01 | -0.03 | 0.08 | -0.18 |
| ΔA peak velocity, cm/s | 0.12 | 0.04 | 0.03 | 0.05 | -0.07 |
| ΔE/A ratio | 0.02 | 0.13 | 0.10 | 0.03 | -0.14 |
| ΔE/E' ratio | 0.12 | 0.10 | 0.11 | -0.24 | -0.04 |
| ΔLV end-diastolic volume, ml | 0.15 | 0.11 | 0.08 | 0.04 | 0.04 |
| ΔLV end-systolic volume, ml | 0.03 | 0.03 | -0.05 | 0.11 | 0.07 |
| ΔLV ejection fraction, % | 0.10 | 0.14 | 0.09 | 0.14 | 0.10 |
| ΔLV stroke volume, ml | 0.20 | 0.13 | 0.16 | -0.06 | 0.04 |
| ΔCardiac output, ml/min ^\|\|^ | 0.15 | 0.03 | 0.08 | -0.16 | 0.08 |
| ΔGlobal longitudinal strain, % ^n=48^ | 0.14 | 0.11 | 0.16 | -0.07 | 0.07 |
| ΔGLS exercise 25W ^n=28^ | 0.01 | -0.08 | 0.05 | -0.13 | -0.11 |
| ΔGLS exercise 50W ^n=27^ | 0.05 | -0.01 | 0.02 | 0.02 | 0.12 |
| ΔGLS exercise 75W ^n=27^ | **-0.41*** | -0.35 | -0.30 | -0.01 | -0.31 |
| ΔGLS exercise 100W ^n=19^ | 0.15 | 0.24 | 0.28 | 0.03 | -0.05 |
| ΔGLS exercise 125W ^n=11^ | 0.01 | -0.04 | -0.04 | 0.24 | 0.52 |

Hb = Hemoglobin; HCT = Hematocrit; RBC = red blood cell; MVC = mean corpuscular volume; MCH = mean corpuscular hemoglobin; BMI = body mass index; LPA = light-intensity physical activity; MVPA = moderate-to-vigorous physical activity; VO_2_max = maximal oxygen consumption; FFM = fat free mass; VO_2_ = oxygen consumption; RER = respiratory exchange ratio; HOMA-IR = Homeostatic Model Assessment for Insulin Resistance; M-value = whole-body glucose uptake in hyperinsulinemic euglycemic clamp (HEC); LDL = low density lipoprotein; HDL = high density lipoprotein; ALT = alanine aminotransferase; AST = aspartate aminotransferase; GGT = γ-glutamyltransferase; LV = Left ventricle; LA = left atrium; E = peak early diastolic filling velocity; A = peak late (atrial) diastolic filling velocity; E’ = lateral early diastolic mitral annulus velocity; GLS = global longitudinal strain.

Partial correlation, controlling for BMI, was performed.
Bold values indicate statistical significance: * = p < 0.05, ** = p < 0.01, ***=p < 0.001.

†=n=40; ‡=n=43; §=n=44; ||=n=49; ¶=n=58; ††=n=59; ‡‡ = 60 ; §§=n=62; ||||=n=63.
